# PI3K**γ** promotes neutrophil extracellular trap formation by noncanonical pyroptosis in abdominal aortic aneurysm

**DOI:** 10.1172/jci.insight.183237

**Published:** 2024-07-18

**Authors:** Yacheng Xiong, Shuai Liu, Yu Liu, Jiani Zhao, Jinjian Sun, Yongqing Li, Baihong Pan, Wei Wang

**Affiliations:** 1 Department of General & Vascular Surgery, Xiangya Hospital, Central South University, Changsha, China.; 2 Department of Surgery, University of Michigan Health System, Ann Arbor, Michigan, USA.; 3 National Clinical Research Center for Geriatric Disorders, Xiangya Hospital, Central South University, Changsha, China.

**Keywords:** Vascular biology, Drug therapy, Neutrophils

## Abstract

Abdominal aortic aneurysm (AAA) is one of the most life-threatening cardiovascular diseases; however, effective drug treatments are still lacking. The formation of neutrophil extracellular traps (NETs) has been shown to be a crucial trigger of AAA, and identifying upstream regulatory targets is thus key to discovering therapeutic agents for AAA. We revealed that phosphoinositide-3-kinase γ (PI3Kγ) acted as an upstream regulatory molecule and that PI3Kγ inhibition reduced NET formation and aortic wall inflammation, thereby markedly ameliorating AAA. However, the mechanism of NET formation regulated by PI3Kγ remains unclear. In this study, we showed that PI3Kγ deficiency inactivated the noncanonical pyroptosis pathway, which suppressed downstream NET formation. In addition, PI3Kγ regulation of noncanonical pyroptosis was dependent on cyclic AMP/protein kinase A signaling. These results clarify the molecular mechanism and crosstalk between PI3Kγ and NETosis in the development of AAA, potentially facilitating the discovery of therapeutic options for AAA.

## Abstract 2

PI3Kgamma deficiency inhibits the noncanonical pyroptosis pathway via cAMP/PKA signaling, thereby reducing NETs formation, alleviating abdominal aortic aneurysm.

## Graphical abstract

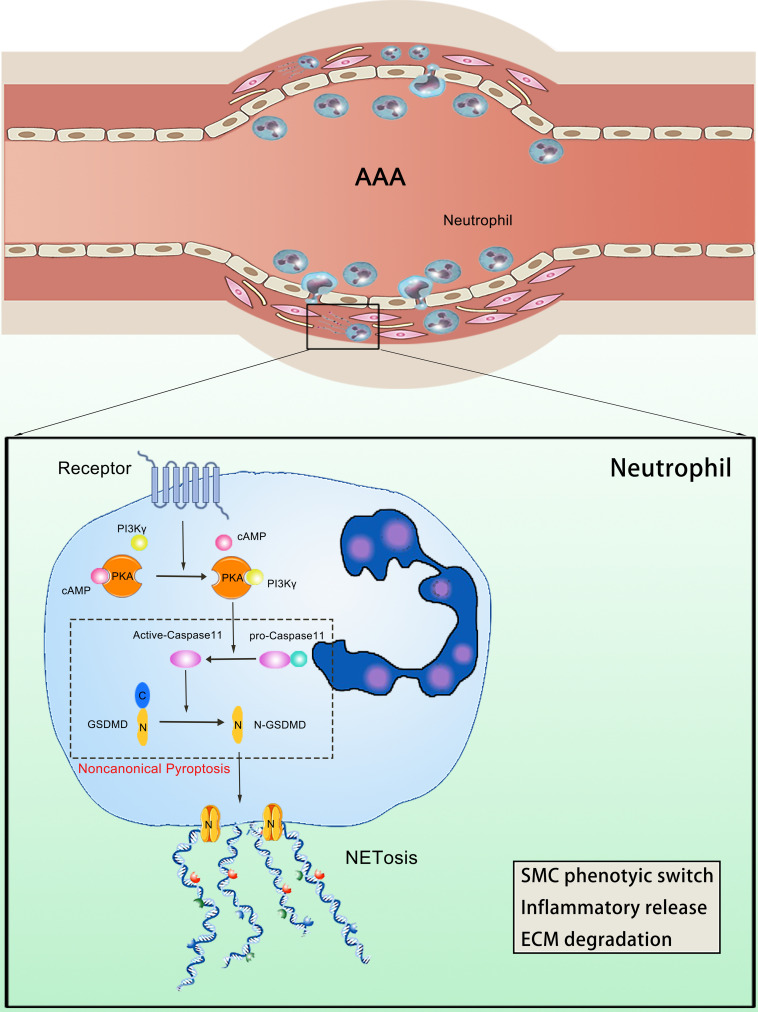

## Introduction

Abdominal aortic aneurysm (AAA) is a balloon-like bulge in the abdominal segment of the aortic wall, caused by various factors, and its rupture is a major cause of death in adults (1). The prevalence of AAA is up to 0.92% in people aged 30–79 worldwide (2). Progressive aneurysm dilatation eventually progresses to aneurysm rupture, with a mortality rate of more than 81% (3). To date, there is no effective drug treatment to prevent the formation and growth of aneurysms (4), and existing drugs used in clinical trials for the treatment of AAA have not yet shown good efficacy (5). There is thus an urgent need to explore the potential mechanisms responsible for AAA and to discover effective therapies.

Inflammation plays a pivotal role in the pathological process of AAA (6). Various immune cells, including neutrophils, monocytes/macrophages, eosinophils, and lymphocytes, infiltrate the arterial wall tissue, which is associated with the development of AAA (7). Among these inflammatory cells, neutrophils are recruited to the inflammatory vessel wall at an early stage of AAA formation (8). A prospective cohort study showed a strong positive association between neutrophil count and AAA development, independent of other traditional risk factors, such as smoking, obesity, and atherosclerosis (9). Limiting neutrophil recruitment to the arterial wall reduced the incidence of elastase perfusion–induced AAA (10), and inhibition of neutrophils using anti-neutrophil antibodies also limits the progression of experimental AAA (8). In addition, neutrophil-derived biomarkers may be of clinical value for the monitoring and prognosis of AAA and may be used to guide early therapeutic interventions (11), suggesting that neutrophils may be a therapeutic target in AAA. The application of neutrophil-neutralizing antibodies to reduce neutrophils in the treatment of AAA, however, has only a short-term effect and damages the host’s innate immune defense function (12). Elucidating the mechanism of neutrophils promoting AAA is therefore important for the discovery of therapeutic drug targets.

Neutrophils play an important role in immune defense through phagocytosis, degranulation, and formation of neutrophil extracellular traps (NETs) (13). NETs are composed of depolymerized chromatin, citrullinated histone 3 (Cit H3), granule proteins, and cytoplasmic proteins (14). NET formation contributes to pathogen clearance; however, uncontrolled NET formation accelerates the deterioration of sterile inflammatory diseases, including atherosclerosis (15). Previous studies showed that circulating NETs were strongly correlated with the severity of AAA (16) and that NETs aggregated AAA via the initiation and promotion of inflammatory responses, smooth muscle phenotype switching, and extracellular matrix degradation through matrix metalloproteinases (MMPs) (17–19). A further study demonstrated that reducing NET formation in the abdominal aorta markedly protected against elastase-induced AAA (20). Blockage of NET formation is currently achieved through inhibition of peptidyl arginine deiminases (PADs), which are expressed in neutrophils and drive histone citrullination (21). Notably however, PAD-deficient mice exhibited innate immune dysfunction and were more susceptible to bacterial infections (22), indicating the need to identify another possible trigger of NETs for the development of therapeutic agents for AAA (23).

Phosphoinositide-3-kinase γ (PI3Kγ) belongs to the PI3K protein family, which is mainly expressed in immune cells and is involved in the progression of inflammatory diseases. Blocking PI3Kγ is accordingly regarded as an effective strategy for the treatment of inflammatory diseases (24). DeSouza-Vieira et al. reported that the release of NETs from human neutrophils was dependent on the activation of PI3Kγ during Leishmania infection (25). A recent study also revealed that PI3Kγ inhibition could reduce NET formation in the treatment of microscopic polyarteritis (26), suggesting that PI3Kγ may be an upstream regulator of NET formation. However, the role of PI3Kγ in AAA progression via promoting NET formation remains unclear, and the mechanism by which PI3Kγ regulates NETs is still poorly understood.

Herein, we revealed that PI3Kγ inhibition reduced neutrophil infiltration and NET formation in the arterial wall of AAA. Reduction of NET formation by PI3Kγ blockade was achieved by restraining the noncanonical pyroptosis pathway, and PI3Kγ regulated this pathway via cAMP/PKA signaling. We clearly demonstrated that PI3Kγ regulated the progression of AAA via a mechanism involving PI3Kγ→cAMP/PKA→noncanonical pyroptosis→NETosis. The results of this study provide insight into the prevention of AAA and broaden our understanding of the upstream regulatory sites of NET formation.

## Results

### Neutrophil infiltration and NET formation are upregulated in human and mouse AAA tissues.

**Figure 1:**
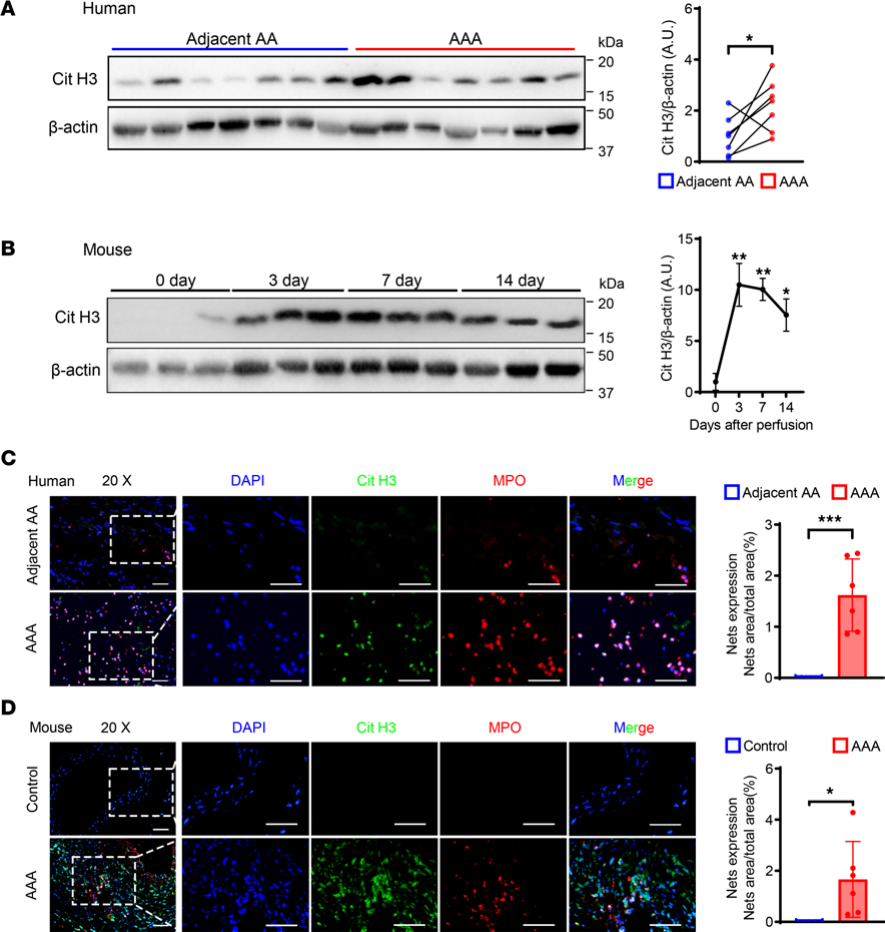
NETs are upregulated in both human and mouse AAA tissue. (**A**) Representative Western blot image and quantitative analysis of Cit H3 protein expression in human AAA tissue and adjacent AA tissue. *n* = 7. (**B**) Representative Western blot image and quantitative analysis of Cit H3 protein expression in mouse AAA tissue at different time points after PPE surgery. *n* = 3. (**C**) Representative immunofluorescence staining images and quantitative comparison of NETs in human abdominal aortic sections. NETs’ expression was calculated by NET area/total area (%) according to the fluorescence colocalization of DNA (DAPI, blue), citrullinated histone 3 (Cit H3, green), and myeloperoxidase (MPO, red). *n* = 6. Scale bars, 50 μm. (**D**) Representative immunofluorescence staining images and quantitative comparison of NETs in mouse abdominal aorta after PPE-induced AAA. NETs’ expression was calculated by NET area/total area (%) according to the fluorescence colocalization of DNA (DAPI, blue), citrullinated histone 3 (Cit H3, green), and myeloperoxidase (MPO, red). *n* = 5 in control group and *n* = 6 in AAA group. Scale bars, 50 μm. (**A**) Two-tailed paired Student’s *t* test. (**B**) One-way ANOVA followed by Dunnett’s test. (**C** and **D**) Two-tailed unpaired Student’s *t* test. *P < 0.05, **P < 0.01, and ***P < 0.001. Adjacent AA, adjacent abdominal aorta; AAA, abdominal aortic aneurysm; Cit H3, citrullinated histone 3; PPE, porcine pancreatic elastase; NETs, neutrophil extracellular traps.

To reveal levels of NET formation in the AAA wall, we quantified NETs in human AAA samples and control adjacent abdominal aortic tissue (adjacent AA). Expression levels of Cit H3 protein, as a marker of NETs, were markedly upregulated in AAA tissues (Figure 1A). Additionally, we constructed porcine pancreatic elastase–induced (PPE-induced) AAA and quantified the timing of NET formation. Protein from abdominal aortas was collected at 0, 3, 7, and 14 days after elastase perfusion. Cit H3 expression peaked in aortas from days 3–7 and was downregulated at day 14 (Figure 1B). Furthermore, we performed immunofluorescence staining and verified the change of NETs’ expression. The results revealed increased NET expression in AAA tissues (Figure 1, C and D). To further reveal neutrophil infiltration in AAA, neutrophils (Ly6G^+^) were evaluated by immunohistochemistry. Neutrophil infiltration was increased in aortas from AAA compared with saline-treated mice (Supplemental Figure 2A; supplemental material available online with this article; https://doi.org/10.1172/jci.insight.183237DS1). These results suggest that abnormal neutrophil infiltration and NET expression were closely associated with AAA.

### Inhibition of NETs alleviates elastase-induced AAA in mice.

**Figure 2:**
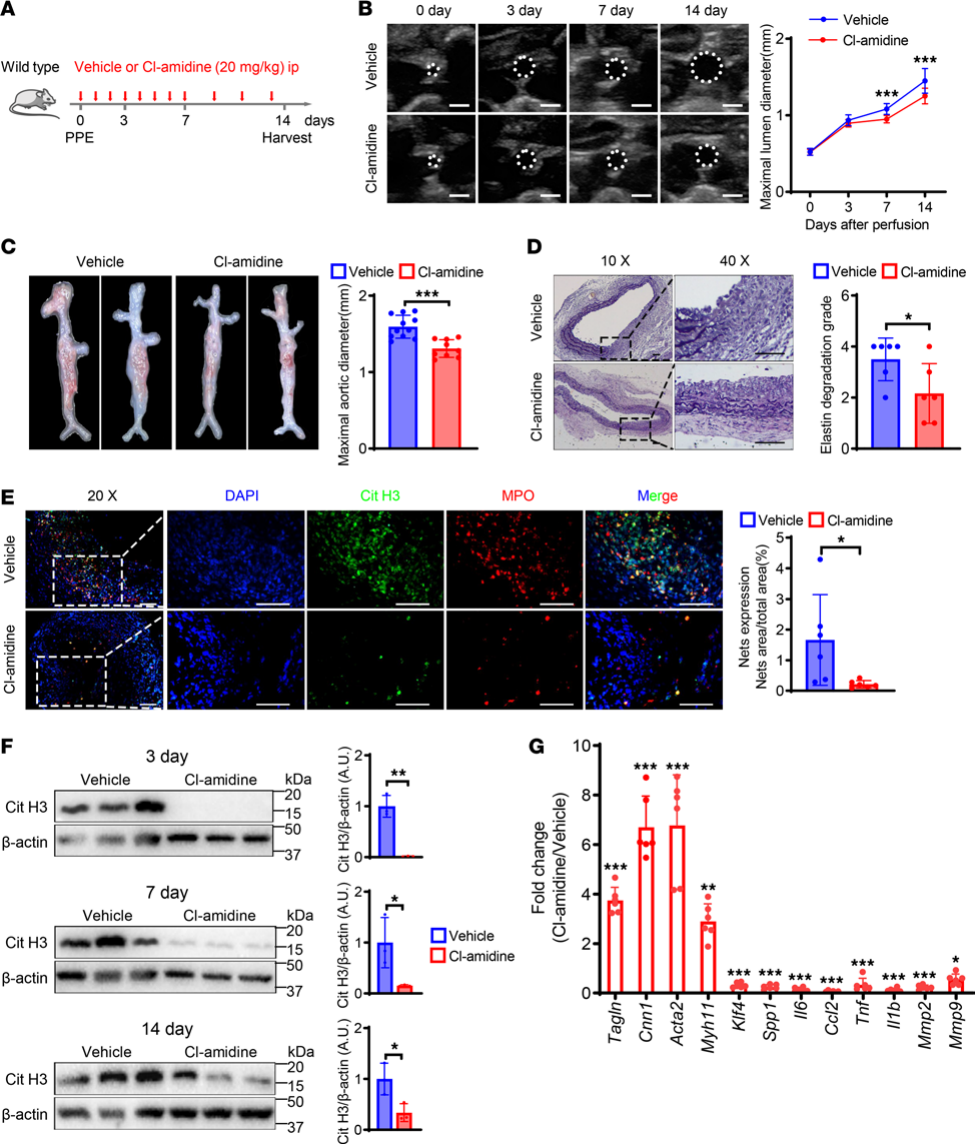
Inhibition of NETs alleviates elastase-induced AAA in mice. (**A**) Schematic diagram of the animal study to verify the effect of Cl-amidine administration on PPE-induced AAA. (**B**) Representative ultrasound images and quantitative comparison of maximal lumen diameter in mouse abdominal aorta at different time points with various treatments. *n* = 13 in vehicle group and *n* = 15 in Cl-amidine group. Scale bar: 1 mm. (**C**) Representative macroscopic images and quantitative comparison of maximal aortic diameter in mouse abdominal aorta at 14 days after PPE-induced AAA. *n* = 11 in vehicle group and *n* = 9 in Cl-amidine group. (**D**) Representative VVG staining images and quantitative comparison of mouse abdominal aorta in PPE-induced AAA. *n* = 6. Scale bars, 50 μm. (**E**) Representative immunofluorescence staining images and quantitative comparison of NETs in mouse abdominal aorta after PPE-induced AAA. NETs’ expression was calculated by NET area/total area (%) according to the fluorescence colocalization of DNA (DAPI, blue), citrullinated histone 3 (Cit H3, green), and myeloperoxidase (MPO, red). *n* = 6. Scale bars, 50 μm. (**F**) Representative Western blot image and quantitative analysis of Cit H3 protein expression in mouse AAA tissue at different time points with various treatment. *n* = 3. (**G**) qPCR analysis of contractile, synthetic, inflammation, and matrix metalloproteinase genes’ expression normalized to the mean expression of housekeeping gene (*Actb*) in mouse abdominal aorta from vehicle and Cl-amidine administration after PPE-induced AAA. *n* = 6. (**B** and **G**) Two-way ANOVA followed by Bonferroni’s test. (**C**–**F**) Two-tailed unpaired Student’s *t* test. *P < 0.05, **P < 0.01, and ***P < 0.001. ip, intraperitoneal injection.

To reveal the crucial effect of NET formation in AAA development, we examined the effect of Cl-amidine (a PAD4 inhibitor, blocking NET formation) on PPE-induced AAA (Figure 2A). Ultrasonography of the abdominal aorta demonstrated smaller aorta lumen diameters in the Cl-amidine group compared with the vehicle (saline) group from days 7–14 after PPE induction surgery (Figure 2B). There were also significant differences in the external diameter of the aorta between the Cl-amidine and vehicle groups (Figure 2C). Histomorphology showed that the degradation of elastin in the Cl-amidine group was less destroyed than in the control group (Figure 2D). To verify the inhibitory effect of Cl-amidine, we carried out immunofluorescence staining of NETs in the aorta and showed that NET expression was markedly decreased after Cl-amidine administration (Figure 2E). In addition, Cit H3 protein expression was significantly reduced at any time point after PPE surgery in Cl-amidine–treated mice (Figure 2F). Previous studies showed that NETs exacerbated AAA by promoting smooth muscle phenotypic switching, inflammatory release, and matrix degradation (19). We observed similar results. mRNA levels of the vascular smooth muscle cell (VSMC) contractile genes *Tagln*, *Cnn1*, *Acta2*, and *Myh11* were increased while the synthetic genes *Klf4* and *Spp1* were reduced in the Cl-amidine group. In addition, the proinflammatory genes *IL6*, *TNFα*, *Ccl2*, and *IL-1β* and the MMP genes *MMP2* and *MMP9* were significantly decreased in the abdominal aorta of Cl-amidine–treated mice compared with control mice (Figure 2G). These data indicate that blocking the formation of NETs effectively inhibited the progression of AAA.

### PI3Kγ knockout reduces neutrophil infiltration and NETs’ release in AAA.

**Figure 3:**
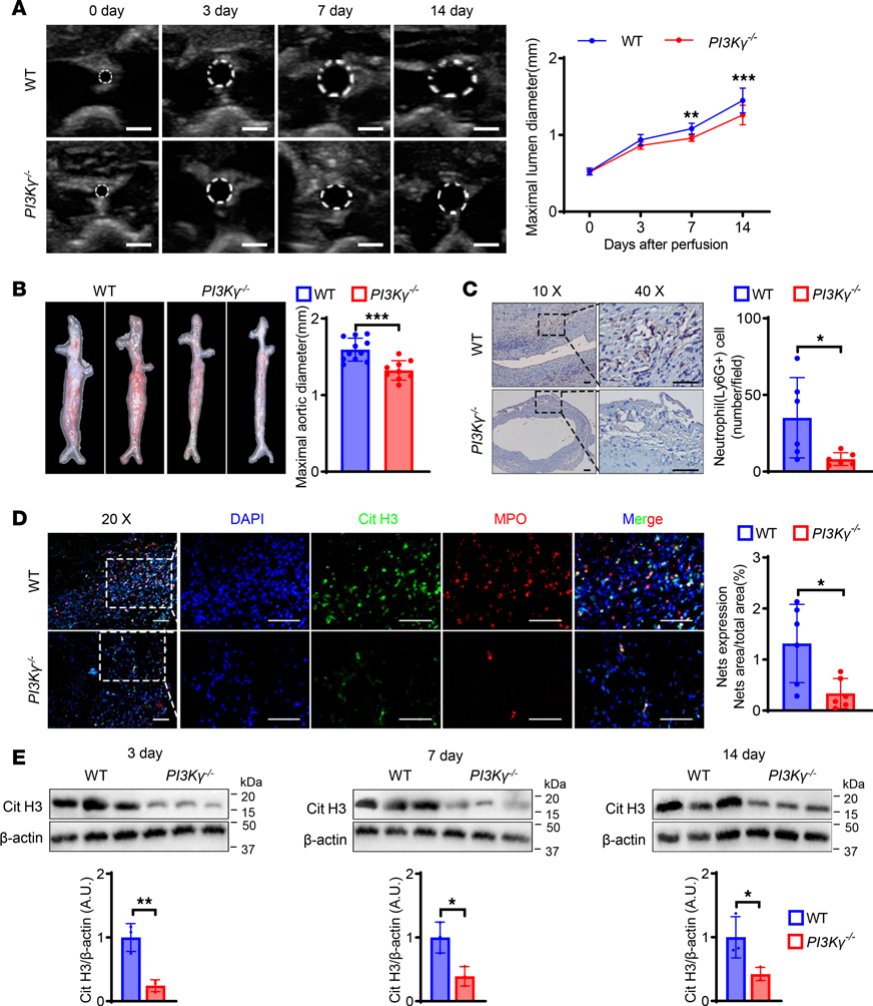
PI3Kγ knockout reduces neutrophil infiltration and NET formation and improves AAA. (**A**) Representative ultrasound images and quantitative comparison of maximal lumen diameter in mouse abdominal aorta at different time points after PPE-induced AAA. *n* = 13. Scale bar: 1 mm. (**B**) Representative macroscopic images and quantitative comparison of maximal aortic diameter in mouse abdominal aorta at 14 days after PPE-induced AAA. *n* = 11 in WT group and *n* = 9 in *PI3Kγ^–/–^* group. (**C**) Representative immunohistochemistry staining images and quantitative comparison of neutrophil infiltration in mouse abdominal aorta after PPE-induced AAA. Neutrophil infiltration was assessed by Ly6G^+^ neutrophil numbers per high-power field (original magnification, 40×). *n* = 6. Scale bars, 50 μm. (**D**) Representative immunofluorescence staining images and quantitative comparison of NETs in mouse abdominal aorta after PPE-induced AAA. NETs’ expression was calculated by NET area/total area (%) according to the fluorescence colocalization of DNA (DAPI, blue), citrullinated histone 3 (Cit H3, green), and myeloperoxidase (MPO, red). *n* = 6. Scale bars, 50 μm. (**E**) Representative Western blot image and quantitative analysis of Cit H3 protein expression in mouse AAA tissue at different time points after PPE surgery. *n* = 3. (**A**) Two-way ANOVA followed by Bonferroni’s test. (**B**–**E**) Two-tailed unpaired Student’s *t* test. *P < 0.05, **P < 0.01, and ***P < 0.001. *PI3Kγ^–/–^*, phosphoinositide-3-kinase γ knockout.

Given that inhibition of PAD impairs the innate immune defense system (22), we explored upstream regulatory factors of NET formation. PI3Kγ is involved in the regulation of neutrophil function (27), and we speculated that PI3Kγ might participate in the development of AAA via inducing NET formation. We therefore generated PI3Kγ-knockout mice (*PI3Kγ^–/–^*) (Supplemental Figure 1, A and B). PI3Kγ protein expression level was significantly increased in the abdominal aorta in WT mice on day 14 after elastase perfusion compared with day 0 (Supplemental Figure 1C). To further elucidate the impact of PI3Kγ on AAA, we induced AAA in *PI3Kγ^–/–^* and WT mice and showed that PI3Kγ deficiency alleviated PPE-induced AAA, similar to Cl-amidine administration. Ultrasonography, gross morphology, and elastic Van Gieson staining of the abdominal aorta demonstrated that *PI3Kγ^–/–^* mice had less destroyed aortas compared with WT mice (Figure 3, A and B, and Supplemental Figure 2B). These results suggested that PI3Kγ was an important etiological factor of AAA. Meanwhile, neutrophil infiltration and NET formation were reduced in the *PI3Kγ^–/–^* group compared with the WT group (Figure 3, C and D), and Cit H3 protein expression was significantly lower in *PI3Kγ^–/–^* compared with WT mice (Figure 3E). These results suggested that PI3Kγ acted as an upstream regulatory molecule of NET formation.

We further determined the downstream signals of NETs by real-time quantitative PCR. There were no significant differences in the VSMC markers *Cnn1*, *Acta2*, and *Klf4* between the WT and *PI3Kγ^–/–^* groups; however, there were significant differences in *Tagln*, *Myh11*, and *Spp1* between the 2 groups. The inflammatory genes, *IL6*, *TNFa*, *Ccl2*, and *IL-1β*, were significantly downregulated in *PI3Kγ^–/–^* mice, and MMP9, but not MMP2, expression levels were also reduced in *PI3Kγ*^–/–^ mice (Supplemental Figure 2C). All these results suggest that PI3Kγ blockade may alleviate the development of AAA by reducing the formation of NETs.

### Deficiency of PI3Kγ inhibits NETs’ formation in neutrophils.

**Figure 4:**
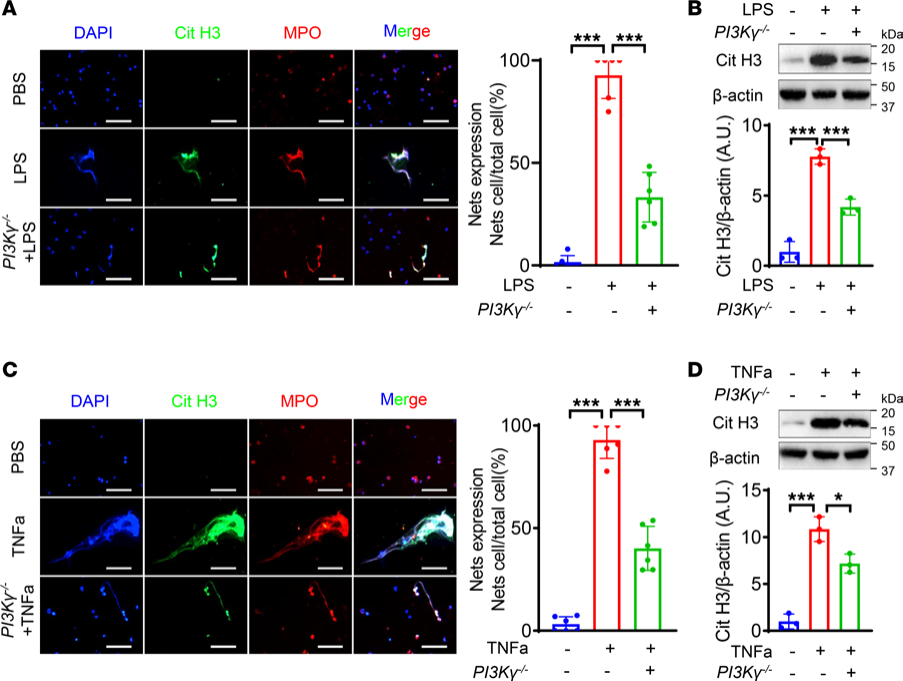
Deficiency of PI3Kγ inhibits NETs’ formation in neutrophils. (**A** and **C**) Representative immunofluorescence staining images and quantitative comparison of NETs produced by neutrophils with different treatments: PBS (WT-derived neutrophils), LPS (5 μg/mL, WT-derived neutrophils), LPS (*PI3Kγ^–/–^*–derived neutrophils), PBS (WT-derived neutrophils), TNF-α (50 ng/mL, WT-derived neutrophils), TNF-α (*PI3K*γ*^–/–^*–derived neutrophils). NETs were detected using immunofluorescence staining of DNA (DAPI, blue), citrullinated histone 3 (Cit H3, green), and myeloperoxidase (MPO, red). NETs’ expression was calculated by NET-expressing cell numbers/total cell numbers per high-power field (original magnification, 40×). *n* = 6. Scale bars, 50 μm. (**B** and **D**) Representative Western blot images and quantitative comparison of Cit H3 protein expression in each group of neutrophils with different treatments as described in **A** and **C**. *n* = 3. (**A**–**D**) One-way ANOVA followed by Fisher’s least significant difference post hoc test. *P < 0.05, and ***P < 0.001. PBS, phosphate-buffered saline.

To clarify the causal relationship between PI3Kγ and NET formation in neutrophils, we isolated and purified neutrophils (purity > 90%) from mice (Supplemental Figure 3, A–C), and stimulated them with LPS, as a classical inducer of NETs (28). The most appropriate concentration of LPS to induce NETs was 5 μg/mL (Supplemental Figure 3D). LPS-treated neutrophils from WT mice formed large numbers of NETs, and LPS-induced NET formation was significantly inhibited by the PI3Kγ inhibitor (IPI549) (Supplemental Figure 4, A and B). We also mimicked the inflammatory state of AAA by stimulation of isolated neutrophils with TNF-α. NET formation was significantly increased by TNF-α but was reversed after IPI549 administration (Supplemental Figure 4, C and D). We further corroborated the experimental results, by repeating most of the above experiments in neutrophils purified from *PI3Kγ^–/–^* and WT mice, and showed consistent results with those following PI3Kγ inhibitor treatment (Figure 4, A–D). All these results suggested that inhibition of PI3Kγ in neutrophils could reduce NET formation in vitro.

### PI3Kγ expression in neutrophils was required for NET formation and AAA progression in mice.

**Figure 5:**
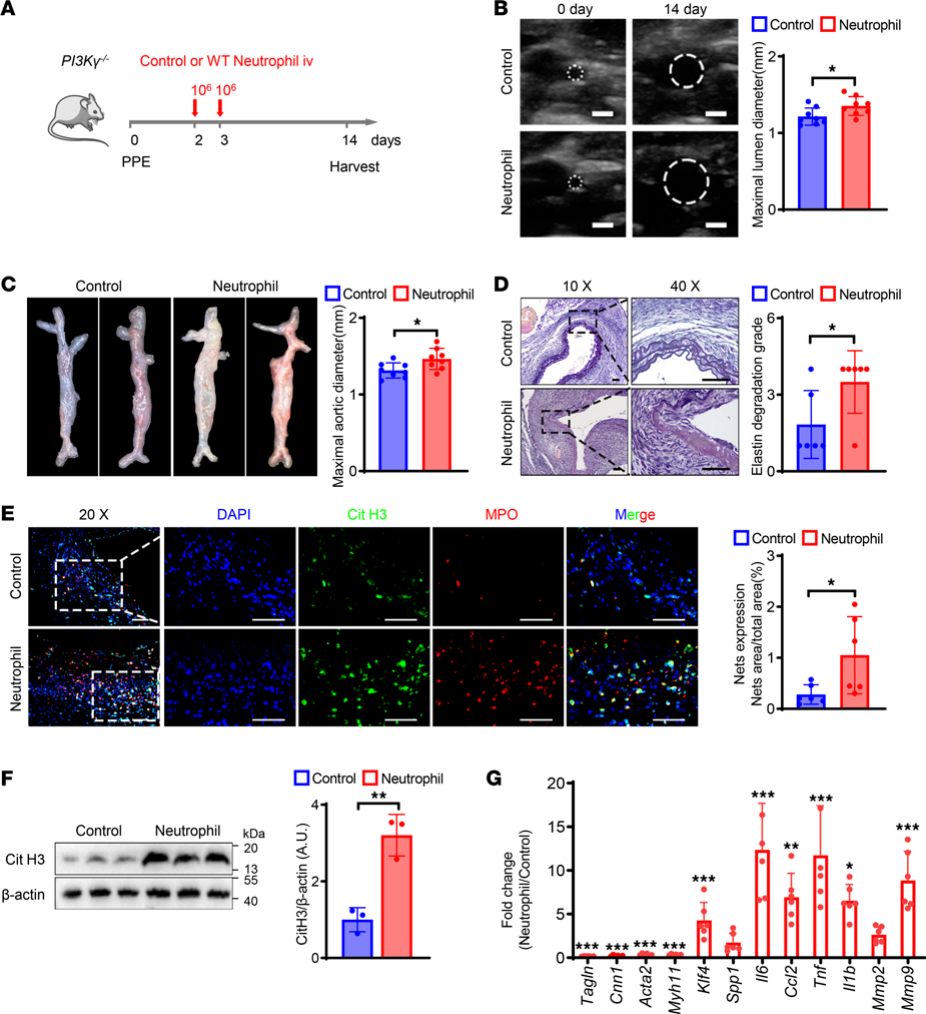
PI3Kγ expression in neutrophils is required for NETs’ formation and AAA progression in mice. (**A**) Schematic diagram of the experimental process of neutrophil adoptive transfer in PPE-induced AAA *PI3Kγ^–/–^* mice. (**B**) Representative ultrasound images and quantitative comparison of maximal lumen diameter in *PI3Kγ*^–/–^ mice after PPE surgery. *n* = 8. Scale bar: 1 mm. (**C**) Representative macroscopic images and quantitative comparison of maximal aortic diameter in *PI3Kγ^–/–^* mice after PPE surgery. *n* = 8. (**D**) Representative VVG staining images and quantitative comparison of VVG staining score in *PI3Kγ^–/–^* mice after PPE surgery. *n* = 6. Scale bars, 50 μm. (**E**) Representative immunofluorescence staining images and quantitative comparison of NETs in *PI3Kγ^–/–^* mouse abdominal aorta after PPE-induced AAA. NETs’ expression was calculated by NET area/total area (%) according to the fluorescence colocalization of DNA (DAPI, blue), citrullinated histone 3 (Cit H3, green), and myeloperoxidase (MPO, red). *n* = 6. Scale bars, 50 μm. (**F**) Representative Western blot image and quantitative analysis of Cit H3 protein expression in the abdominal aorta of *PI3Kγ^–/–^* mice. *n* = 3. (**G**) qPCR analysis of contractile, synthetic, inflammation, and matrix metalloproteinase genes expression normalized to the mean expression of housekeeping gene (*Actb*) in abdominal aorta of *PI3Kγ^–/–^* mice from control and WT neutrophil adoptive transfer after PPE-induced AAA. *n* = 6. (**B**–**F**) Two-tailed unpaired Student’s *t* test. (**G**) Two-way ANOVA followed by Bonferroni’s test. *P < 0.05, **P < 0.01, and ***P < 0.001. iv, intravenous injection; VVG, Verhoeff Van Gieson.

To further reveal the role of PI3Kγ from neutrophils in AAA formation, we generated elastase-induced AAA in *PI3Kγ^–/–^* mice. Neutrophils from WT mice are the only source of PI3Kγ in *PI3Kγ^–/–^* mice, and we therefore performed adoptive transfer of neutrophils isolated from WT mice into *PI3Kγ^–/–^* mice. A schematic diagram of the process is shown in Figure 5A. *PI3Kγ^–/–^* mice that received WT neutrophils exhibited more severe AAA lesions compared with *PI3Kγ^–/–^* mice without neutrophil transfer. Both the maximal lumen and external diameters of the abdominal aorta were significantly greater in neutrophil-transferred *PI3Kγ^–/–^* mice than in vehicle-treated *PI3Kγ^–/–^* mice (Figure 5, B and C). Verhoeff Van Gieson (VVG) staining of the abdominal aorta showed more severe elastin degradation in neutrophil-transferred *PI3Kγ^–/–^* mice (Figure 5D). We also evaluated NET formation by immunofluorescence and Western blot and showed that NET expression was significantly increased in neutrophil-transferred *PI3Kγ^–/–^* compared with vehicle-treated *PI3Kγ^–/–^* mice (Figure 5, E and F). Furthermore, adoptive transfer of neutrophils significantly decreased the expression of the VSMC contractile genes *Acta2*, *Cnn1*, *Tagln*, and *Myh11* and significantly increased the expression of genes associated with the synthetic and inflammatory phenotype and extracellular matrix degradation (Figure 5G). These results suggest that neutrophil-derived PI3Kγ acted as a key regulatory molecule in NET formation in a mouse model of PPE-induced AAA.

### PI3Kγ promotes NET formation via noncanonical pyroptosis pathways in vitro.

The above results suggested that inhibition of PI3Kγ alleviated AAA by suppressing NETosis. We therefore investigated the molecular mechanism by which PI3Kγ regulated NET formation. Previous studies identified ROS as a critical factor in the induction of NET formation (29). We therefore detected the protein expression of NADPH oxidase 2 (NOX2, a key enzyme in inducing ROS production) by Western blot. LPS-treated neutrophils released large numbers of NETs, whereas PI3Kγ inhibition or administration of the specific NOX inhibitor, diphenyleneiodonium chloride (DPI), significantly reduced NET formation, with an obvious synergistic effect. Interestingly however, PI3Kγ blockage had no effect on NOX2 protein expression (Supplemental Figure 5, A and B). These results suggest that the inhibitory effect of PI3Kγ deficiency on NET formation in neutrophils was independent of ROS signaling.

**Figure 6:**
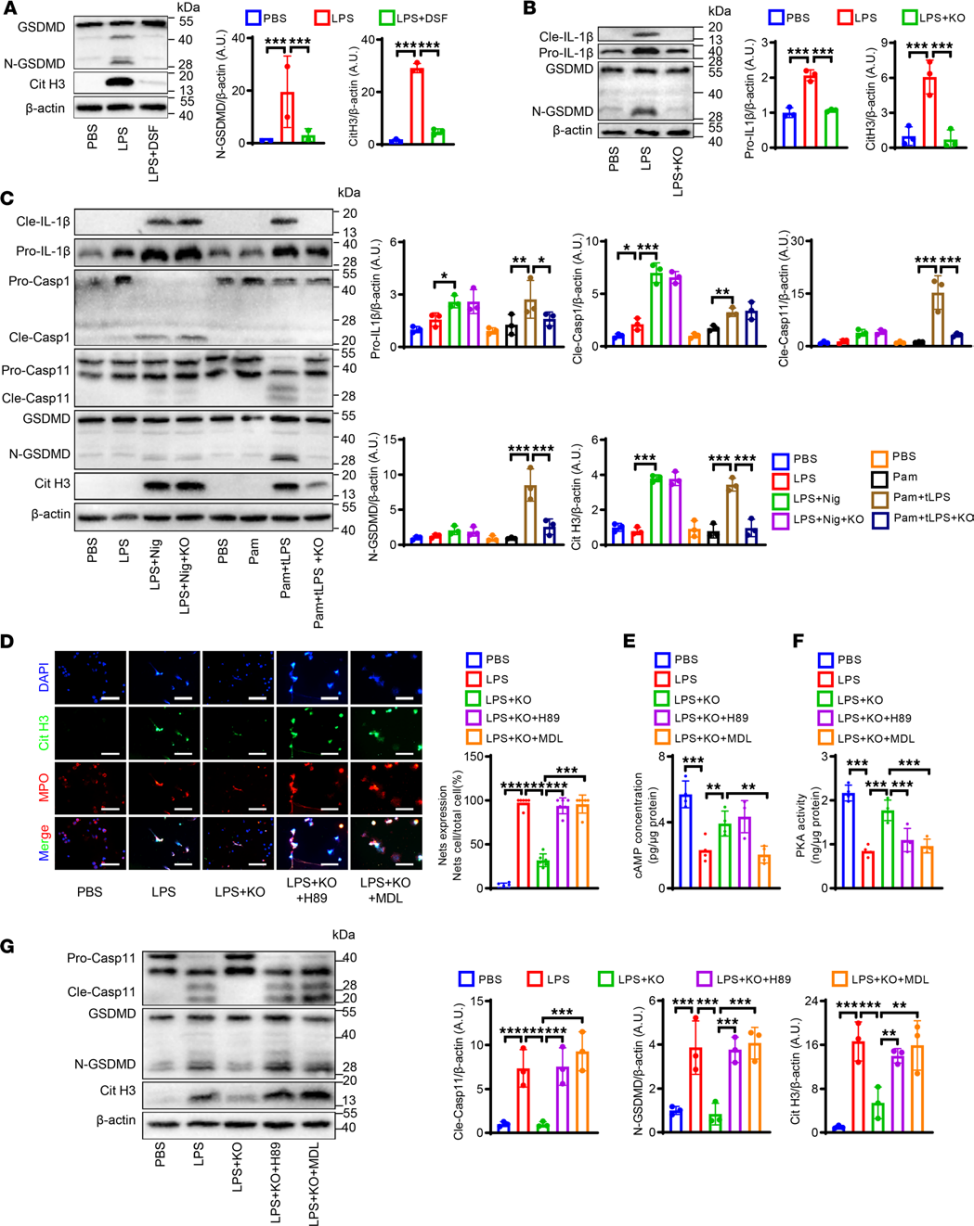
PI3Kγ promotes NETs’ formation via noncanonical pyroptosis pathways in vitro. (**A**) Representative Western blot (**A**) and quantitative comparison of Cit H3 and GSDMD in WT neutrophils with different treatments: PBS, LPS (5 μg/mL), and LPS + DSF (30 μM). *n* = 3. (**B**) Representative Western blot and quantitative comparison of IL-1β and GSDMD in neutrophils with different treatments: PBS, LPS (5 μg/mL), LPS + KO (*PI3Kγ^–/–^*). *n* = 3. (**C**) Representative Western blot and quantitative comparison of Cit H3, IL-1β, GSDMD, caspase-11, and caspase-1 in the neutrophils with different treatments: PBS, LPS (100 ng/mL), LPS + nigericin (10 μM), LPS + nigericin + KO, PBS, Pam3CSK4 (1 μg/mL), Pam3CSK4 + transfer LPS (tLPS, 10 μg/mL), Pam3CSK4 + tLPS + KO. *n* = 3. (**D**) Representative immunofluorescence staining and quantitative comparison of NETs produced by neutrophils with different treatments: PBS, LPS (5 μg/mL), LPS + KO, LPS + H89 (20 μM) + KO, LPS + MDL12330A (10 μM) + KO. NETs were detected using immunofluorescence staining. NETs’ expression was calculated by NET-expressing cell numbers/total cell numbers per high-power field (original magnification, 40×). *n* = 6. Scale bars, 50 μm. (**E**) Comparison of cAMP concentration of neutrophils with different treatments as described in **D** by ELISA. *n* = 4. (**F**) Comparison of PKA kinase activity of neutrophils with different treatments as described in **D** by kinase activity assay. *n* = 4. (**G**) Representative Western blot and quantitative comparison of Cit H3, GSDMD, and caspase-11 in the neutrophils with different treatments as described in **D**. *n* = 3. (**A**–**G**) One-way ANOVA followed by Fisher’s least significant difference post hoc test. *P < 0.05, **P < 0.01, and ***P < 0.001. GSDMD, gasdermin D; pro-, prosoma; cle-, cleaved; Casp1, caspase-1; Casp11, caspase-11; LPS, lipopolysaccharide; DSF, disulfiram; Nig, nigericin; Pam, Pam3CSK4; KO, phosphoinositide-3-kinase γ knockout; MDL, MDL12330A.

Previous studies also suggested that pyroptosis may be involved in the formation of NETs (30). We therefore measured the expression levels of gasdermin D (GSDMD, a key protein in the pyroptosis signaling pathway). Protein levels of N-GSDMD, the active splicing form of GSDMD, were significantly increased in human AAA compared with adjacent AA tissue (Supplemental Figure 6A). WT mouse neutrophils were isolated and treated with LPS. LPS treatment significantly increased N-GSDMD and Cit H3 protein levels, and these effects were reversed by the specific GSDMD inhibitor disulfiram (DSF) (Figure 6A). These results indicate that pyroptosis may be a key upstream regulatory signal for NET formation.

To investigate if PI3Kγ regulated NET formation via the pyroptosis pathway, we examined the expression of pyroptosis pathway–related proteins in WT and *PI3Kγ^–/–^* neutrophils. IL-1β and N-GSDMD expression levels were significantly inhibited in PI3Kγ-deficient mice (Figure 6B). These findings suggested that pyroptosis was a key bridge for PI3Kγ to promote NET formation. Previous studies showed that the occurrence of pyroptosis depends on the activation of the canonical or noncanonical inflammatory pathway (31). We therefore examined the involvement of canonical and noncanonical inflammatory pathways in PI3Kγ-induced NET formation. To activate the canonical inflammatory pathway, neutrophils from WT or *PI3Kγ^–/–^* mice were primed with LPS followed by administration of nigericin to activate NLRP3 inflammasomes (caspase-1), while noncanonical inflammatory pathways were activated using the TLR1/2 agonist Pam3CSK4 to suppress basal apoptosis, followed by LPS transfection into the neutrophil cytosol to activate caspase-11. Both canonical and noncanonical inflammasome activation significantly increased Cit H3 expression and IL-1β release; however, only caspase-11/GSDMD signaling (noncanonical pyroptosis pathway) was inhibited after PI3Kγ blockade, whereas there was no significant difference in canonical pyroptosis pathway–associated proteins (caspase-1/IL-1β) between *PI3Kγ*^–/–^ and WT groups (Figure 6C). We then repeated the above experiment in neutrophils isolated from WT mice using a PI3Kγ inhibitor (IPI549) and showed that the results were consistent with those from PI3Kγ-derived neutrophils (Supplemental Figure 6C). These results suggested that PI3Kγ might promote NET formation via noncanonical pyroptosis pathways.

We then explored the molecular mechanism by which blocking PI3Kγ inhibited the nonclassical pyroptosis pathway. The most common pathway is one by which PI3Kγ may directly regulate PI3K/Akt signaling through its lipid kinase function, which is thought to be directly downstream of PI3Kγ. We therefore detected NET expression after treatment with the specific Akt agonist SC79. PI3Kγ blockade reduced Akt phosphorylation and NET formation. Interestingly, phosphorylation of Akt was increased, but Cit H3 expression was decreased after SC79 treatment (Supplemental Figure 7, A and B). These results suggested that PI3Kγ regulated noncanonical pyroptosis independent of Akt, indicating an alternative signaling pathway. PI3Kγ can function as an anchor protein (32) for PKA to regulate the cAMP/PKA signaling pathway in a negative feedback loop (33). We speculated that PKA signaling might be involved in PI3Kγ-mediated regulation of the noncanonical pyroptosis pathway. We therefore blocked PKA signaling using a PKA inhibitor, H89, and an adenylyl cyclase inhibitor/cAMP product inhibitor, MDL12330A. Decreased NET expression by PI3Kγ blockade was counteracted by H89 or MDL12330A (Figure 6D). We further evaluated the cAMP/PKA signaling pathway by ELISA and kinase activity assays. PI3Kγ inhibition reversed LPS-induced activation of cAMP/PKA signaling, while H89 and MDL12330A attenuated these processes (Figure 6, E and F). Concomitantly, Western blot analysis showed that PI3Kγ deficiency deactivated the LPS-induced caspase-11/GSDMD signaling pathway. Notably, PI3Kγ-mediated inhibition of noncanonical pyroptosis in neutrophils was markedly reversed by pretreatment with H89 or MDL12330A (Figure 6G). Similarly, these results were recapitulated in IPI549-treated neutrophils from WT mice (Supplemental Figure 8, A and B). Together, these data indicate that PI3Kγ may regulate noncanonical pyroptosis through the cAMP/PKA signaling pathway.

### cAMP/PKA inhibitor eliminates the protective effect of PI3Kγ knockout in elastase-induced AAA.

**Figure 7:**
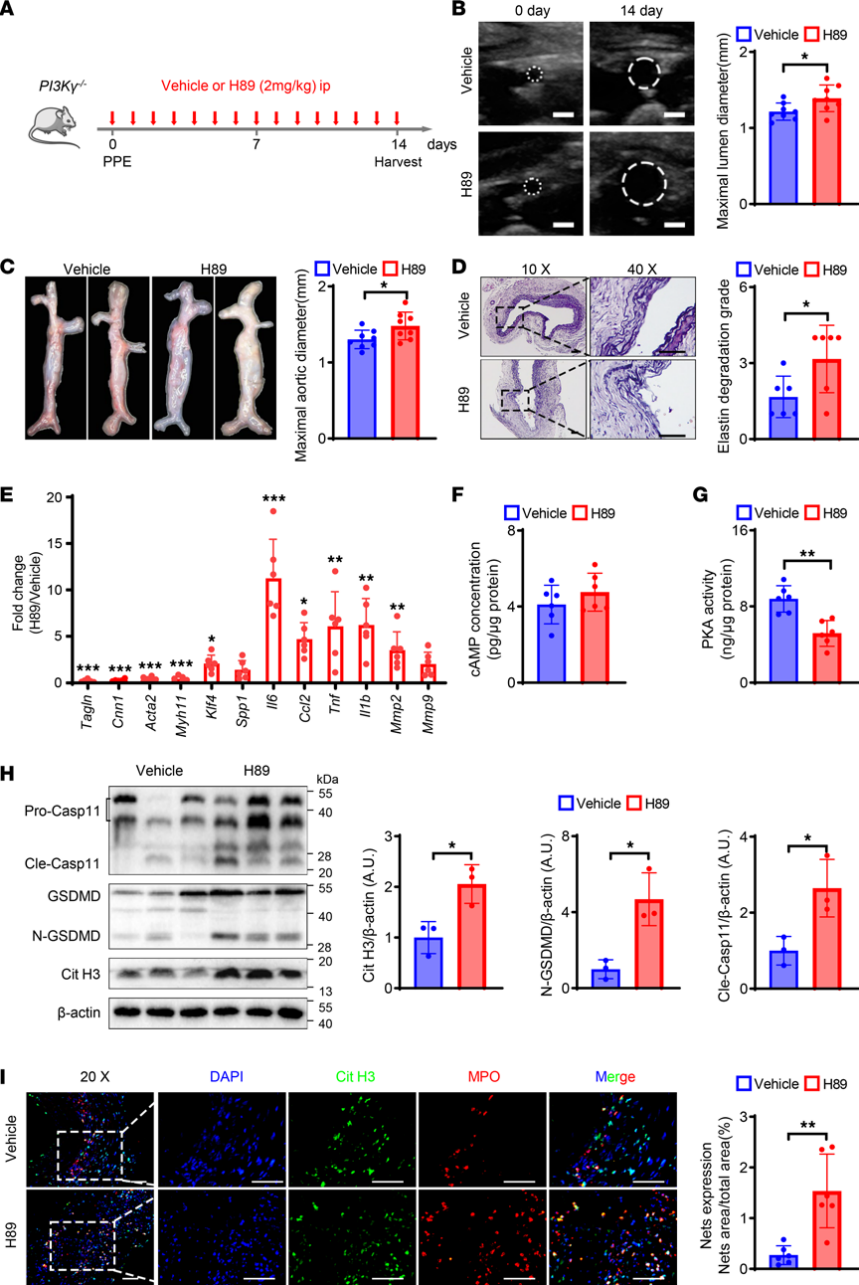
cAMP/PKA inhibitor eliminates protective effect of PI3Kγ knockout in elastase-induced AAA. (**A**) Schematic diagram of the animal study to verify the effect of H89 administration in PPE-induced AAA *PI3Kγ^–/–^* mice. (**B**) Representative ultrasound images and quantitative comparison of maximal lumen diameter in PI3K*γ*^–/–^ mice after PPE surgery. *n* = 8. Scale bar: 1 mm. (**C**) Representative macroscopic images and quantitative comparison of maximal aortic diameter in *PI3Kγ*^–/–^ mice after PPE surgery. *n* = 8. (**D**) Representative VVG staining images and quantitative comparison of VVG staining score in *PI3Kγ^–/–^* mice after PPE surgery. *n* = 6. Scale bars, 50 μm. (**E**) qPCR analysis of contractile, synthetic, inflammation, and matrix metalloproteinase genes’ expression normalized to the mean expression of housekeeping gene (*Actb*) in abdominal aorta of *PI3Kγ^–/–^* mice from vehicle and H89 administration after PPE-induced AAA. *n* = 6. (**F** and **G**) The concentration of cAMP (**F**) and PKA activity (**G**) in the abdominal aorta of *PI3Kγ^–/–^* mice. *n* = 6. (**H**) Representative Western blot image and quantitative analysis of Cit H3, GSDMD, and caspase-11 protein expression in the abdominal aorta of *PI3Kγ^–/–^* mice. *n* = 3. (**I**) Representative immunofluorescence staining images and quantitative comparison of NETs in *PI3Kγ^–/–^* mouse abdominal aorta after PPE-induced AAA. NETs’ expression was calculated by NET area/total area (%) according to the fluorescence colocalization of DNA (DAPI, blue), citrullinated histone 3 (Cit H3, green), and myeloperoxidase (MPO, red). *n* = 6. Scale bars, 50 μm. (**B**–**D** and **F**–**I**) Two-tailed unpaired Student’s *t* test. (**E**) Two-way ANOVA followed by Bonferroni’s test. *P < 0.05, **P < 0.01, and ***P < 0.001.

To investigate the possible involvement of cAMP/PKA signaling in AAA, *PI3Kγ^–/–^* mice received PPE-induced AAA and H89. H89 treatment significantly increased the AAA size compared with vehicle treatment (Figure 7, A–C), and elastin fragmentation in the aortic wall was more severe in the H89 group compared with the vehicle group (Figure 7D). In addition, H89 significantly decreased the mRNA levels of *Acta2*, *Cnn1*, *Tagln*, and *Myh11* in the abdominal aorta and increased the mRNA levels of *Klf4*, as well as *IL6*, *TNFα*, *Ccl2*, *IL-1β*, and *MMP2* (Figure 7E). Additionally, we detected the cAMP concentration and PKA activity. H89 treatment significantly inhibited PKA kinase activity, with no obvious increase in cAMP levels (Figure 7, F and G). Immunofluorescence and Western blot results showed a significant increase in the expression of NETs following H89 administration, compared with the vehicle group (Figure 7, H and I). Moreover, H89 aggravated caspase-11 activation and GSDMD cleavage in the arterial wall of *PI3Kγ^–/–^* mice (Figure 7I). These results suggested that PI3Kγ deficiency suppressed noncanonical pyroptosis and thus inhibited NET formation in a cAMP/PKA-dependent manner.

## Discussion

Robust infiltration of inflammatory cells plays a critical role in the pathogenesis of AAA. Among all inflammatory cells, NETs derived from neutrophils are a key trigger inducing AAA dilation (6). Targeting NET formation may thus represent an avenue for treating AAA. Here we revealed that PI3Kγ, as the upstream regulatory molecule of NETs’ formation, may be a potential target for inhibiting AAA progression. Meanwhile, we elucidated that the molecular mechanism by which PI3Kγ regulated NET release was dependent on noncanonical pyroptosis. These findings provide a theoretical basis for targeting PI3Kγ to reduce arterial wall inflammation and treat AAA.

A previous study found that NETs induced VSMC apoptosis through p38/JNK signaling, degraded elastic fibers, and promoted angiotensin II–induced (Ang II–induced) AAA (34). Another recent study showed that circulating NETs were positively correlated with clinical outcomes of AAA; NETs promoted VSMC inflammatory release and phenotypic transformation through Hippo/Yap signaling activation, exacerbating AAA dilation (17). These publications suggest that NETs play a critical role in promoting AAA progression. We also found a marked increase in NET formation in human AAA and elastase-induced AAA, consistent with these previous findings. We further determined the expression of NETs at different time points in a mouse model of elastase-induced AAA and showed that NETs peaked at days 3–7, then decreased by day 14 after PPE surgery. This was consistent with previous studies (35, 36) showing that neutrophils were the initiating cells of the inflammatory response, and infiltration of the arterial wall occurred in the early stage of aneurysm formation, which may explain the decrease in NETs in the late stage of aneurysm.

To date, studies on NET formation and aneurysms remain scarce. Although several studies have revealed a causal relationship between NETs and AAA (17, 34, 37), they mainly focused on the downstream mechanism by which NETs promoted AAA formation, while upstream regulatory mechanisms of NETs’ formation were rarely discussed, which may provide a key target for therapeutic drug discovery. A recent study reported that treatment with the PAD4 inhibitor GSK484 inhibited NET formation and attenuated Ang II–induced experimental aneurysm progression (16). We subsequently investigated the protective effect of the specific PAD4 inhibitor Cl-amidine on aneurysms, and the results were consistent with previous studies (20), suggesting that NET formation played a crucial role in AAA formation. Unfortunately, the application of PAD inhibitors targeting NET formation will damage the innate immune function (22). They are thus not suitable for prolonged administration for the treatment of AAA. It is therefore essential to identify upstream regulators of NETs for AAA treatment. Previous studies showed that inhibition of PI3Kγ promoted neutrophil senescence and reduced NETs, which in turn improved acute lung injury (38). In addition, PI3Kγ depletion protected against microscopic polyangiitis by inhibiting NET formation (26). We hypothesized that PI3Kγ may be involved in AAA progression by affecting NET formation. We accordingly showed that PI3Kγ was markedly upregulated in elastase-induced AAA mice, and PI3Kγ knockout markedly inhibited the formation of AAA. Furthermore, PI3Kγ blockade dramatically suppressed neutrophil infiltration and NET formation, inhibiting AAA formation. These results suggest that PI3Kγ may be an upstream regulatory molecule of NETs in AAA formation and progression.

Although we used PI3Kγ–global knockout mice to demonstrate a protective effect of PI3Kγ in AAA, it is difficult to explain that PI3Kγ of neutrophils plays a key role in AAA progression. PI3Kγ is widely expressed in many inflammatory cell types (39). Previous studies suggested that PI3Kγ promoted carotid re-endothelialization and aggravated vascular stenosis through Cxcl10 secretion by Th1 cells (40). PI3Kγ facilitated LDL uptake in macrophages to form foam cells and exacerbated atherosclerotic plaque formation (41). Therefore, PI3Kγ may also promote the release of NETs and aggravate AAA through other inflammatory cells. To clarify whether it is neutrophil-derived PI3Kγ that regulates NETs’ release and promotes AAA, we isolated neutrophils from mouse bone marrow and showed that inhibition of PI3Kγ reduced NET release, consistent with previous studies (42). Subsequently, we adoptively transferred bone marrow neutrophils from WT mice to *PI3Kγ*^–/–^ mice to clarify the contribution of PI3Kγ in neutrophils to elastase-induced AAA and showed that adoptively transferred WT neutrophils markedly worsened AAA in *PI3Kγ*^–/–^ mice, verifying the involvement of neutrophil-derived PI3Kγ in aneurysms.

Despite the fact that PI3Kγ is involved in regulating NET formation during AAA, the molecular mechanism is still poorly understood. Previous studies identified ROS as an important inducer regulating the formation of NETs (43) and NOX2 as a key enzyme that produces ROS to mediate the formation of NETs (44). However, our data showed that PI3Kγ blockade reduced the expression of NETs, but did not decrease the upregulation of NOX2 upon LPS stimulation, and had a synergistic effect when combined with a NOX2 inhibitor. This suggested that PI3Kγ regulated NET expression independent of ROS signaling pathways. Recent studies showed that GSDMD, as a key executioner of pyroptosis, was required for the formation of NETs (30). Cleaved GSDMD enhanced nuclear membrane permeability at the early stage of NET formation, thus facilitating the entry of cytoplasmic granules into the nucleus followed by pore formation at the cytoplasmic membrane to promote NET complex release (45), indicating that NET formation was closely related to pyroptosis. The FDA-approved drug DSF, which is used to treat alcohol addiction, was recently shown to inhibit GSDMD pore formation (46). We found that LPS-induced NET formation was markedly reduced after DSF administration, consistent with previous studies (47). We therefore hypothesized that PI3Kγ regulated NET expression through pyroptosis. Accordingly, pyroptosis-related N-GSDMD was markedly increased in human AAA, validating the clinical relevance of pyroptosis in AAA. In addition, pyroptosis signaling was markedly reduced under PI3Kγ deficiency, revealing its involvement in the regulation of pyroptosis. This provides a report of a link between PI3Kγ and pyroptosis.

Notably however, the mechanism of the effects of PI3Kγ on pyroptosis is poorly understood. Previous studies showed that the induction of pyroptosis could be mediated by canonical and noncanonical pyroptosis pathways. Canonical pyroptosis results from the activation of caspase-1 by the inflammatory complex, whereas noncanonical pyroptosis results from the activation of caspase-11 by intracellular LPS (31). We therefore induced canonical and noncanonical pyroptosis using LPS followed by nigericin stimulation, and Pam3CSK4 followed by LPS transfection, respectively, according to the previous publications (48). PI3Kγ deficiency reduced the formation of NETs and inhibited the activation of noncanonical pyroptosis and the release of IL-1β but had no effect on the canonical pyroptosis pathway. These results suggest that PI3Kγ regulates NET formation via noncanonical pyroptosis.

We further explored the mechanism by which PI3Kγ regulates noncanonical pyroptosis. PI3Kγ has phosphoinositide kinase activity, which phosphorylates phosphatidylinositol-2-phosphate to phosphatidylinositol-3-phosphate, leading to the recruitment of Akt with a Pleckstrin homology structural domain and activation of downstream signaling. Previous studies indicated that PI3Kγ-driven NET formation was dependent on the activation of Akt signaling (38, 42). However, they did not perform rescue experiments to prove that PI3Kγ regulated NET formation through PI3K/Akt. Our results suggested that PI3Kγ blockade–reduced NET formation was accompanied by inhibition of Akt signaling, consistent with previous results. In contrast, when Akt signaling was markedly activated by SC79, NET levels were further reduced, implying that blocking PI3Kγ and Akt activation could synergistically suppress NET formation. A recent study suggested that activation of the AKT/mTOR autophagy pathway promoted the transformation of neutrophil function from NETosis to phagocytosis (49). This may explain the protective effect on NETs’ formation after Akt signaling activation. The above results indicated that PI3Kγ regulated NETs in a manner independent of Akt signaling.

Patrucco et al. found that PI3Kγ, in addition to phosphatidyl kinase activity, acted as an anchoring protein with a bridging function, negatively regulating cardiac contractility (50). PI3Kγ promoted the interaction of PKA with phosphodiesterase and negatively regulated cAMP/PKA signaling (51). We thus hypothesized that PI3Kγ regulated neutrophil noncanonical pyroptosis through cAMP/PKA signaling. We accordingly showed that the cAMP/PKA signaling, which was activated by PI3Kγ deletion, was resuppressed in neutrophils after treatment with H89 and MDL12330A, and the protective effect of PI3Kγ deletion on NET formation was abolished. Additionally, the inhibitory effect of PI3Kγ deficiency on noncanonical pyroptosis signaling was also reversed after H89 and MDL12330A administration. We further explored the role of cAMP/PKA signaling in PI3Kγ-mediated AAA within elastase-induced AAA and showed that blocking PKA signaling abrogated the protective effect of PI3Kγ deficiency on AAA, while NETs’ formation and noncanonical pyroptosis signaling in the arterial wall were also markedly enhanced. These results explain the molecular mechanism by which PI3Kγ regulates noncanonical pyroptosis–mediated NET formation in AAA.

This study had certain limitations. One limitation is that although we showed that PI3Kγ inhibition reduced NET formation, the precise molecular mechanism of NET formation remains unclear. Several different stimulus-specific signaling pathways are involved in NETosis, and further investigations should be performed to determine the potential crosstalk between the various upstream signaling pathways of NETosis, especially in AAA. The second limitation is that our goal in this study is to elucidate the role of neutrophilic PI3Kγ on AAA development while PI3Kγ–global knockout mice were applied in this experiment; neutrophil-specific PI3Kγ knockout is more suitable, though a neutrophil transfer experiment can support our conclusion. The third limitation is that previous studies have shown that endothelial PI3Kγ deficiency reduced neutrophil infiltration (52), which suggested that PI3Kγ derived from other cells may also play important functions in AAA. Therefore, a neutrophil- or endothelium-specific PI3Kγ-knockout model should be used to further investigate and support the functions of PI3Kγ in AAA development.

In conclusion, our results revealed the molecular mechanism by which PI3Kγ regulates NET formation. We showed that PI3Kγ blockade markedly inhibited neutrophil infiltration and NET formation in the abdominal aorta, notably ameliorating multiple pathological changes in AAA. Previous studies have reported serious adverse events of current FDA-approved PI3K inhibitors (53), which may compromise their clinical usage. However, our study demonstrates that the PI3Kγ-specific inhibition or downstream signaling pathway (for example NETosis) may be a therapeutic target for AAA treatment with fewer side effects. The results of this study may thus promote PI3Kγ-specific inhibitors as potentially useful candidate drugs for the treatment of AAA.

## Methods

### Sex as a biological variable.

Human aorta samples were collected. Men and women were involved. The AAA animal model was performed and only male mice were used. Sex was not considered as a biological variable.

### Reagents.

The key reagents used in this experiment are shown in Supplemental Table 1.

### Study design, participant recruitment, and sample collection.

In the present study, patients with AAA were recruited at Xiangya Hospital (Changsha, China) from September 2021 to October 2022. AAA was confirmed by computed tomography angiography according to the 2018 American Society for Vascular Surgery guidelines for the diagnosis and treatment of AAA (54). AAA samples were collected from patients receiving open aneurysm repair. Control tissue samples were derived from adjacent, relatively normal abdominal aorta tissue. The demographic and clinical details of the patients are shown in Supplemental Table 2. All included participants provided written informed consent.

### Animal study.

Male WT specific pathogen–free (SPF) C57BL/6J mice aged 8 weeks were purchased from SPF Hunan SJA Laboratory Animal Co., Ltd. (Changsha, China). *PI3K*γ*^–/–^* mice were purchased from GemPharmatech Co. Ltd. (Nanjing, China). The construction strategy and genotype identification are shown in Supplemental Figure 1, A and B.

The PPE-induced AAA model was established by intravascular PPE perfusion as described previously (55). Briefly, mice were anesthetized with 1.5% isoflurane, and PPE (4.0 U/mL dissolved in sterile saline) was delivered into the infrarenal aorta for 5 minutes at 150 mmHg. Control mice underwent sham surgery and were administered an identical volume of saline.

Cl-amidine (20 mg/kg) and H89 (2 mg/kg) were administered by intraperitoneal injection in PPE-induced AAA model mice at different endpoints.

Neutrophil adoptive transfer mice were established by tail vein injection of neutrophils (10^6^/mouse) once a day on days 2–3 after creation of the PPE models, as described previously (20). The AAA model was established in PI3Kγ-knockout mice. The experimental group mice were injected with neutrophils from WT mice, and the control group mice were injected with neutrophils from PI3Kγ-knockout mice. The number of neutrophils injected was consistent.

All AAA model mice were sacrificed for measurement of the maximal aortic diameter and tissue collection on day 14 after surgery. All animals were housed in SPF conditions at 23 ± 1°C and 50%–60% humidity on a 12-hour light/12-hour dark cycle and were provided with a standard chow diet and water ad libitum.

### Blood count.

Mouse blood samples were collected and analyzed using an automated hematology analyzer (BC2800vet, Mindray) (Supplemental Figure 1D).

### ELISA.

Mouse blood samples were collected and centrifuged at 500*g* for 5 minutes at 4°C. Serum was collected and stored at −80°C. Serum inflammatory factor concentrations were measured using a Mouse ELISA Kit in accordance with the manufacturer’s instructions (Supplemental Figure 1E).

### Aortic ultrasound imaging.

Abdominal aortas were visualized in isoflurane-anesthetized mice at the indicated endpoints using a color Doppler ultrasound system with ZS3 Exp Domain scanning imaging technique. Cross-sectional images of the abdominal aorta were captured, and the maximal lumen diameters at the aneurysm sites were measured. All measurements were made by 2 researchers in a blinded manner.

### Neutrophil isolation and culture.

Mice were sacrificed and bone marrow was removed. Neutrophils were isolated from the bone marrow using a mouse marrow neutrophil isolation kit (Solarbio, P8550). Immediately after isolation, the neutrophil layer was collected and resuspended in RPMI 1640 medium containing 5% fetal bovine serum and 1% penicillin/streptomycin. The cells were incubated at 37°C and 5% CO_2_ in a humidified incubator and used for NETosis studies after 30 minutes of isolation. The concentration of neutrophils was identified by staining and flow cytometry (Supplemental Figure 3, A–C).

### Induction of NETs and interventions in vitro.

Freshly purified neutrophils were grown on poly-l-lysine–coated coverslips in a 24-well plate. NETosis was induced by different concentrations of LPS, with an optimal concentration of 5 μg/mL (Supplemental Figure 3D). Neutrophils were treated with PBS as a negative control and LPS (5 μg/mL, MilliporeSigma) or TNF-α (50 ng/mL, MedChemExpress) for 4 hours to induce NET formation.

For NETosis, neutrophils were pretreated with IPI549 (5 μM, MedChemExpress) or DSF (30 μM, MedChemExpress) for 1 hour and then stimulated with LPS (5 μg/mL, MilliporeSigma) for 4 hours to induce NET formation. In addition, to interfere with the ROS or Akt pathways, neutrophils were pretreated with DPI (10 μM, MedChemExpress) or SC79 (4 μg/mL, MedChemExpress) for 1 hour after IPI549 administration, then stimulated with LPS for 4 hours to induce NET formation.

To explore pyroptosis during NETosis, neutrophils were primed with ultrapure LPS (100 ng/mL) for 3 hours, then stimulated with nigericin (10 μM) for 1 hour after IPI549 administration to induce canonical inflammasome activation. For noncanonical inflammasome activation, neutrophils were primed with Pam3CSK4 (1 μg/mL) for 3 hours before IPI549 administration and subsequently transfected without (Mock) or with ultrapure LPS (10 μg/mL) into the cytosol using DOTAP Liposomal Transfection Reagent (Roche) for 4 hours.

For cAMP/PKA inhibition, H89 (20 μM, MedChemExpress) or MDL12330A (10 μM, MedChemExpress) was added to neutrophils for 1 hour after IPI549 administration, followed by stimulation with LPS for 4 hours to induce NET formation.

After stimulation, cells were collected for subsequent tests.

### Immunohistochemistry.

Aorta tissues were fixed in 4% paraformaldehyde (PFA), dehydrated in 70%–100% ethanol, embedded in paraffin, and sectioned (5 μm). Antigen retrieval was performed using sodium citrate–EDTA buffer in an autoclave for 6 minutes, followed by blocking of nonspecific binding with 3% bovine serum albumin (BSA) containing 0.3% Triton X-100 for 1 hour. Tissue sections were incubated with primary antibodies (see Supplemental Table 1) overnight at 4°C. The sections were then rinsed and incubated with HRP-conjugated secondary antibodies from a kit (ZSGB-BIO, PV9000) according to the manufacturer’s protocol, then stained with DAB (ZSGB-BIO, ZLI-9018) and hematoxylin. Images were obtained using a DM6 M microscope (Leica Microsystems).

### Immunofluorescence staining.

Immunofluorescence staining before primary antibody incubation was carried out as for immunohistochemistry. Cells were fixed with 4% PFA for 15 minutes, washed with PBS, and permeabilized with 0.3% Triton X-100 for 10 minutes. After blocking with 3% BSA for 1 hour, tissue sections or cells were incubated with primary antibodies overnight at 4°C followed by corresponding fluorescence-conjugated secondary antibodies for 1 hour at room temperature. The antibodies used are listed in Supplemental Table 1. Images were obtained using Leica DMi8 M. The cells were then stained with DAPI, Cit H3 antibody, and MPO antibody to detect NETs.

### VVG staining.

Aorta tissues were harvested, fixed with 4% PFA, paraffin-embedded, and sectioned (5 μm). The sections were then processed for Verhoeff’s elastic staining using a VVG Elastic Stain Kit (Abiowell, AWI0267b, https://sj.abiowell.com/web/front/cate/product-detail?navCates=3,15,74&id=1659&type=search), according to the manufacturer’s protocol. Images were obtained using a Leica DM6 M microscope. The severity of elastin fragmentation was evaluated and graded as follows: grade 1, no degradation; grade 2, mild elastin degradation; grade 3, moderate elastin degradation; and grade 4, severe elastin degradation. Each sample was assessed by 2 independent pathologists.

### Wright-Giemsa staining.

Purified neutrophils were smeared and stained using Wright-Giemsa stain solution (Solarbio, G1020) in accordance with the manufacturer’s instructions, and images were obtained using a Leica DM6 M.

### Flow cytometry.

Purified neutrophils were resuspended in cell staining buffer and 100 μL of cell suspension (1 × 10^6^ cells/tube), then incubated with Zombie Aqua dye (BioLegend, 423101) for 20 minutes in the dark at room temperature. The cells were then washed with PBS and preincubated with 5 mL of TruStain FcX PLUS (anti-mouse CD16/32) antibody (Fc Receptor Blocking Solution, BioLegend, 156603) for 10 minutes on ice to reduce nonspecific immunofluorescence staining. The cell suspension was then incubated with FITC-conjugated fluorescence antibody against CD11b (BioLegend, 101205) and APC-conjugated fluorescence antibody against Ly6G (BioLegend, 127613) on ice for 20 minutes in the dark (Supplemental Table 1). After 2 washes with PBS, flow cytometry was conducted using a DxP Athena flow cytometer (Cytek Biosciences), and the data were analyzed using FlowJo 10 software.

### cAMP concentration.

cAMP concentrations in tissues and neutrophils were measured using a Mouse cAMP ELISA Kit (Jianglai Biotech, JL13362), in accordance with the manufacturer’s instructions.

### PKA activity.

Purified neutrophil and tissue PKA activities were measured using a nonradioactive PKA Kinase Activity Assay Kit (Abcam, ab139435) following the manufacturer’s instructions.

### Quantitative real-time PCR.

Total RNA was extracted using RNAiso Plus reagent (Takara, 9019) and reverse-transcribed into cDNA using the Hifair AdvanceFast One-step RT-gDNA Digestion SuperMix (Yeasen, 11151ES60). DNA was amplified under the following reaction conditions: denaturing at 95°C for 3 minutes, followed by 40 cycles of annealing at 95°C for 5 seconds and extension at 60°C for 31 seconds in the presence of 2× Universal SYBR Green Fast qPCR Mix (Abclonal, RK21203) and primers. The PCR products were detected using an Applied Biosystems ViiA 7 Real-Time PCR System (Thermo Fisher Scientific). Relative gene expression was determined by the 2^–ΔΔCt^ method. Primers were designed according to the coding sequences of the genes in the National Center for Biotechnology Information database and are listed in Supplemental Table 3.

### Western blot.

Cleaved IL-1β in the cell supernatant was detected after purifying the proteins by the methanol-chloroform extraction method, as described previously (56). Briefly, neutrophil suspensions were centrifuged at 2,000*g* for 5 minutes, the cell supernatant was collected, and 500 μL methanol and 125 μL chloroform were added into 500 μL of supernatant and mixed vigorously. After centrifugation at 16,200*g* for 5 minutes, the upper clear liquid was removed, and 500 μL of methanol was added to the pellet to disperse it. Following another round of centrifugation at 16,200*g* for 5 minutes, the white pellet was left to dry at 55°C for 10 minutes until no visible liquid was evident. Finally, 1× SDS loading buffer was added to the dry proteins and heated for 5 minutes at 95°C, followed by immunoblotting analyses. For cell or tissue samples, proteins were isolated using RIPA buffer (Beyotime, P0013B) containing protease and phosphatase inhibitor (Beyotime, P1045) on ice, and their concentrations were determined using a BCA protein assay kit (Beyotime, P0012). After adding SDS-PAGE protein loading buffer (Beyotime, P0015L) and denaturing at 95°C for 10 minutes, equal amounts of protein samples were separated in an SDS-PAGE gel and transferred to a PVDF membrane. The membrane was blocked with 5% nonfat milk in TBS with Tween 20 buffer for 1 hour at room temperature and incubated with appropriate primary antibodies overnight at 4°C, followed by HRP-conjugated secondary antibody for 1 hour at room temperature. The protein bands were visualized using an Immobilon Western HRP substrate Luminol Reagent (MilliporeSigma, WBKLS0500) and ChemiDoc XRS+ Imaging system (Bio-Rad). Densitometry analysis was performed using ImageJ software (NIH). The antibodies used in the present study are listed in Supplemental Table 1.

### Statistics.

Statistical analysis was performed using Prism 9.5 software (GraphPad Software, Inc.). Values are shown as mean ± SD. Results were compared between 2 groups using 2-tailed unpaired or paired Student’s *t* test and among multiple groups using 1-way ANOVA. Multiple groups with 2 variables were evaluated by 2-way ANOVA followed by Bonferroni’s test. Different interventions were compared among multiple groups using 1-way ANOVA followed by Fisher’s least significant difference post hoc test. For all statistical methods, *P* < 0.05 was considered significant.

### Study approval.

This study was approved by the Ethics Committee of Central South University Xiangya Hospital (Changsha, Hunan province, China, Approval No.201803481) in accordance with the principles of the Declaration of Helsinki. All included participants provided written informed consent. All animal procedures adhered to the US NIH *Guide for the Care and Use of Laboratory Animals* (National Academies Press, 2011).

### Data availability.

The underlying data can be accessed in the Supporting Data Values XLS file.

## Author contributions

BP, Y Li, and WW designed the study and revised the paper. YX performed experiments and wrote the paper. SL, JZ, and JS helped with animal experiments. Y Liu contributed to sample collection.

## Supplementary Material

Supplemental data

Supporting data values

## Version 1 07/18/2024

In-Press Preview

## Version 2 08/22/2024

Electronic publication
