# Supplementary material for: PI3Kγ promotes neutrophil extracellular trap formation by noncanonical pyroptosis in abdominal aortic aneurysm
Source: JCI Insight. 2024 Jul 18;9(16):e183237. doi: 10.1172/jci.insight.183237 (PMC11343590; doi:10.1172/jci.insight.183237)
Supplement: Supplemental data [file jciinsight-9-183237-s117.pdf]

Supplemental information

PI3Kgamma promotes neutrophil extracellular trap formation by noncanonical pyroptosis in abdominal aortic aneurysm

Yacheng Xiong*, Shuai Liu, Yu Liu, Jiani Zhao, Jinjian Sun, Yongqing Li, Baihong Pan#, Wei Wang#

#**Corresponding to**: Wei Wang, weiwangcsu@csu.edu.cn

Baihong Pan, pbh1990s@126.com

This supplementary information includes:

Part I: Supplemental Tables

Table S1: The key reagent and resource used in the present study.

Table S2: The patient clinical information in the present study.

Table S3: The primer sequences used in the present study.

Part II: Supplemental Figures

Figure S1: Construction strategies and identification of PI3Kgamma knockout (*PI3Kγ*$^{-/-}$) mice.

Figure S2: PI3Kγ knockout reduces arterial wall damage and inflammation.

Figure S3: Purity isolated neutrophils from mice marrow stimulated by different

concentration of LPS.

Figure S4: Pharmacological inhibition of PI3Kγ reduces NETs formation in neutrophils.

Figure S5: The inhibitory effect of PI3Kγ deficiency on NET formation in neutrophils is independent of ROS signaling.

Figure S6: Effects of PI3Kγ on the NETs formation of neutrophils via the noncanonical pyroptosis signaling pathways.

Figure S7: The inhibitory effect of PI3Kγ deficiency on NETs formation in neutrophils is independent of PI3K/AKT signaling.

Figure S8: PI3Kγ regulates neutrophil noncanonical pyroptosis via cAMP/PKA signaling pathways.

Supplemental Figures legends

Figure S1. Construction strategies and identification of PI3Kgamma knockout ($PI3K\gamma^{-/-}$) mice.

(A) PI3Kγ knockout mice were generated by cutting exon2-exon9 of the Pik3cg gene using CRISPR/Cas9 technology.

(B) Genotyping was detected by tail preparation of breeding mice.

(C) Representative western blot images showing PI3Kγ protein expression in the aorta at different point time after PPE surgery. n=3.

(D) Blood cell counts of WT and PI3Kγ mice were measured. n=6.

(E) Serum inflammatory factors of WT and PI3Kγ mice were detected. n=6.

WT, wild type; Heter-, heterozygote; Homo-, homozygote. WBC, white blood cell; Lymph, lymphocytes; Mono, monocytes; Gran, granulosa cells; PLT, platelet; IL1B, interleukin-1β; IL6, interleukin-6; TNFA, Tumor Necrosis Factor a; CCL2, C-C motif chemokine ligand 2.

Figure S2. PI3Kγ knockout reduces arterial wall damage and inflammation.

(A) Representative immunohistochemistry staining images and quantitative comparison of neutrophil infiltration in WT mouse abdominal aorta after PPE-induced AAA. Neutrophil infiltration was assessed by Ly6G+ neutrophil numbers per high-power field (40X). n=5 in control group and n=6 in AAA group. Scale bars, 50μm.

(B) Representative VVG staining images and quantitative comparison of WT or $PI3K\gamma^{-/-}$ mouse abdominal aorta in PPE-induced AAA. n = 6. Scale bars, 50 μm.

(C) QPCR analysis of contractile, synthetic, inflammation, and matrix metalloproteinase genes expression normalized to the mean expression of housekeeping gene (*Actb*) in mouse abdominal aorta after PPE-induced AAA. n=6.

(A and B) Two-tailed unpaired student's t test. (C) Two-way ANOVA followed by Bonferroni test.

**P*<0.05, ***P*<0.01, and ****P*<0.001. AAA, abdominal aorta aneurysm; WT, wild type; $PI3K\gamma^{-/-}$, phosphoinositide 3-kinase γ knockout.

Figure S3: Purity isolated neutrophils from mice marrow stimulated by different concentration of LPS.

(A) Representative image of the characteristic morphological appearance of isolated neutrophils stained with Wright-Giemsa stain (40X). Scale bars, 50 μm.

(B) Representative image of the characteristic morphological appearance of isolated neutrophils stained with DAPI (40X). Scale bars, 50 μm.

(C) Representative scatter plot of isolated neutrophils analyzed by flow cytometry. Neutrophils were identified by double positive staining for CD11b and Ly6G.

(D) Representative western blot images and quantitative comparison of Cit H3 protein expression in isolated neutrophils stimulated with different concentration of LPS. n=3.

(D) One-way ANOVA followed by Bonferroni test.

**P*<0.05, ***P*<0.01, and ****P*<0.001. Cit H3, citrullinated histone 3; LPS, lipopolysaccharide.

Figure S4: Pharmacological inhibition of PI3Kγ reduces NETs formation in neutrophils.

(A and C) Representative immunofluorescence staining images and quantitative comparison of NETs produced by isolated neutrophils from WT mice with different treatments: PBS, LPS (5μg/mL), LPS + IPI549 (5μM); PBS, TNFa (50ng/mL), TNFa + IPI549 (5μM). NETs were detected using immunofluorescent staining of DNA (DAPI, blue), Citrullinated histone 3 (Cit H3, Green), and Myeloperoxidase (MPO, red). NETs expression was calculated by NETs cell numbers/total cell numbers per high-power field (40X). n=6. Scale bars, 50 μm.

(B and D) Representative western blot images and quantitative comparison of Cit H3 protein expression in each group of neutrophils with different treatments as described

in (A) and (C). n=3.

(A-D) One-way ANOVA followed by Fisher's LSD post hoc test.

**P*<0.05, ***P*<0.01, and ****P*<0.001.PBS, phosphate buffered saline; LPS, Lipopolysaccharide; TNFa, tumor necrosis factor alpha; Cit H3, citrullinated histone 3; NETs, neutrophil extracellular traps.

Figure S5: The inhibitory effect of PI3Kγ deficiency on NET formation in neutrophils is independent of ROS signaling.

(A) Representative western blot images and quantitative comparison of Cit H3 and NOX2 protein expression in the neutrophils with different treatments: PBS (WT-derived neutrophils), LPS (5μg/mL, WT-derived neutrophils), LPS (PI3Kγ-/- derived neutrophil), LPS + DPI (10μM, WT-derived neutrophils), LPS + DPI (PI3Kγ-/--derived neutrophils). n=3.

(B) Representative western blot images and quantitative comparison of Cit H3 and NOX2 protein expression in isolated neutrophils from WT mice with different treatments: PBS, LPS (5μg/mL), LPS + IPI549 (5μM), LPS + DPI (10μM), LPS + DPI + IPI549. n=3.

(A and B) One-way ANOVA followed by Fisher's LSD post hoc test.

**P*<0.05, ***P*<0.01, and ****P*<0.001. LPS, Lipopolysaccharide; $PI3K\gamma^{-/-}$, phosphoinositide 3-kinase γ knockout; DPI, diphenyleneiodonium chloride; Cit H3, citrullinated histone 3; NOX2, nicotinamide adenine dinucleotide phosphate oxidase 2.

Figure S6: Effects of PI3K γ on the NETs formation of neutrophils via the noncanonical pyroptosis signaling pathways.

(A) Representative western blot image and quantitative analysis of GSDMD protein expression in human AAA tissue and adjacent relatively normal abdominal aortic tissue. n = 7.

(B) Representative western blot image and quantitative comparison of the expression of IL-1β and GSDMD in the isolated neutrophils from WT mice with different treatments: PBS, LPS (5μg/mL), LPS + IPI549 (5μM). n=3.

(C) Representative western blot image and quantitative comparison of the expression of Cit H3, IL-1β, GSDMD, Caspase11, and Caspase1 in the isolated neutrophils from WT mice with different treatments: PBS, LPS (100ng/mL), LPS + Nigericin (10μM), LPS + Nigericin + IPI549 (5μM); PBS, Pam3CSK4 (1μg/mL), Pam3CSK4 + tLPS (10μg/mL, transfer LPS), Pam3CSK4 + tLPS + IPI549 (5μM). n=3. Scale bars, 50 μm.

(A) Two-tailed paired student's t test. (B and C) One-way ANOVA followed by Fisher's LSD post hoc test.

**P*<0.05, ***P*<0.01, and ****P*<0.001. Adjacent AA, adjacent abdominal aorta; AAA, abdominal aorta aneurysm; Cit H3, citrullinated histone 3; GSDMD, Gasdermin D; pro-, prosoma; cle-, cleaved; IL-1β, interleukin 1 beta; Casp1, caspase 1; Casp11, caspase11; PBS, phosphate buffered saline; LPS, Lipopolysaccharide; Nig, nigericin; Pam, Pam3CSK4; tLPS, transfer Lipopolysaccharide.

Figure S7: The inhibitory effect of PI3Kγ deficiency on NETs formation in neutrophils

is independent of PI3K/AKT signaling.

(A) Representative western blot images and quantitative comparison of p-AKT, t-AKT and Cit H3 protein expression in the neutrophils with different treatments: PBS (WT-derived neutrophils), LPS (5μg/mL, WT-derived neutrophils), LPS (PI3Kγ-/- derived neutrophil), LPS + SC79 (4μg/mL, PI3Kγ-/- derived neutrophils). n=3.

(B) Representative western blot images and quantitative comparison of p-AKT, t-AKT and Cit H3 protein expression in isolated neutrophils from WT mice with different treatments: PBS, LPS (5μg/mL), LPS + IPI549 (5μM), LPS + IPI549+ SC79 (4μg/mL). n=3.

(A and B) One-way ANOVA followed by Fisher's LSD post hoc test.

**P*<0.05, ***P*<0.01, and ****P*<0.001. LPS, Lipopolysaccharide; $PI3K\gamma^{-/-}$, phosphoinositide 3-kinase γ knockout; Cit H3, citrullinated histone 3.

Figure S8: PI3Kγ regulates neutrophil noncanonical pyroptosis via cAMP/PKA signaling pathways.

(A) Representative western blot image and quantitative comparison of the expression of Cit H3, GSDMD, and Caspase11 in the isolated neutrophils from WT mice with different treatments: PBS, LPS (5μg/mL), LPS + IPI549 (5μM), LPS + IPI549 + H89 (20μM), LPS + IPI549 + MDL12330A (10μM). n=3.

(B) Representative immunofluorescence staining images and quantitative comparison of NETs produced by neutrophils with different treatments as described in (A). NETs were detected using immunofluorescent staining of DNA (DAPI, blue), Citrullinated

histone 3 (Cit H3, Green), and Myeloperoxidase (MPO, red). NETs expression was calculated by NETs cell numbers/total cell numbers per high-power field (40X). n=6. Scale bars, 50 μm.

(A and B) One-way ANOVA followed by Fisher's LSD post hoc test.

*P<0.05, **P<0.01, and ***P<0.001. Cit H3, citrullinated histone 3; GSDMD, Gasdermin D; pro-, prosoma; cle-, cleaved; Casp11, caspase11; PBS, phosphate buffered saline; LPS, Lipopolysaccharide; MDL, MDL12330A; NETs, neutrophil extracellular traps.

Table S1: the key reagent and resource used in the present study.

Reagent or Resource	Source	Identifier
**Antibodies**		
Rabbit anti-Histone H3 (citrulline R2 + R8 + R17)	Abcam	Cat# ab5103
Mouse anti- PI3-kinase p110 gamma	Santa Cruz Biotechnology	Cat# sc-166365
Rabbit anti-GSDMD	Abcam	Cat# ab210070
Rabbit anti-GSDMD	Abcam	Cat# ab219800
Rabbit anti-AKT	Abcam	Cat# ab179463
Rabbit anti-p-AKT	Abcam	Cat# ab192623
Rabbit anti-NOX2	Abclonal	Cat# A12430
Rabbit anti-Ly6G	Abclonal	Cat# A22270
Rabbit anti-IL-1β	Abcam	Cat# ab254360
Rabbit anti-Caspase11	Abcam	Cat# ab180673
Rabbit anti-Caspase1	Abclonal	Cat# A0964
Goat anti-MPO	R&D	Cat# AF3667-SP
Zombie AquaTM dye	Biolegend	Cat# 423101
TruStain FcX™ PLUS (anti-mouse CD16/32) Antibody	Biolegend	Cat# 156603
APC anti-mouse Ly-6G	Biolegend	Cat# 127613
FITC anti-mouse/human CD11b	Biolegend	Cat# 101205
Alexa Fluor 594-conjugated donkey anti-goat IgG	Invitrogen	Cat# A32758
Alexa Fluor 488-conjugated donkey anti-rabbit IgG	Invitrogen	Cat# A32790
Rabbit anti-β-Actin	Abclonal	Cat# AC038
Mouse anti-GAPDH	Abcam	Cat# ab181602
**Chemicals**		
Precision Plus Protein Dual Color Standards	Bio-Rad	Cat# 1610374
Prestained Protein Marker VII	Servicebio	Cat# G2087
100 bp DNA Ladder	Beijing Tsingke Biotech Co.,	Cat# TSJ100-100

	Ltd.	
Sodium citrate-EDTA antigen repair buffer	Beyotime	Cat# P0086
Mouse/Rabbit Enhanced Polymer test system	ZSGB-BIO	Cat# PV9000
DAB Substrate Kit	ZSGB-BIO	Cat# ZLI-9018
LPS-O111:B4	Sigma-Aldrich	Cat# L3024
Porcine pancreatic elastase	Sigma-Aldrich	Cat# E1250
Opti-MEM	Gibco	Cat# 31985070
TNF-alpha	MedChemExpress	Cat# HY-P7058
IPI549	MedChemExpress	Cat# HY-100716
Cl-amidine hydrochloride	MedChemExpress	Cat# HY-100574A
Disulfiram	MedChemExpress	Cat# HY-B0240
Diphenyleneiodonium chloride	MedChemExpress	Cat# HY-100965
Pam3CSK4	MedChemExpress	Cat# HY-1
Nigericin	MedChemExpress	Cat# HY-1
SC79	MedChemExpress	Cat# HY-18749
H89	MedChemExpress	Cat# HY-15979
MDL12330A	MedChemExpress	Cat# HY-1
DOTAP Liposomal Transfection Reagent	Roche	Cat# 11202375001
DAPI Fluoromount-G	Southernbiotech	Cat# 0100-20
10x Poly-L-lysine Solution	Solarbio	Cat# P2100
RNAiso Plus	Takara	Cat# 9109
RIPA Lysate	Beyotime	Cat# P0013B
Protease and phosphatase inhibitor	Beyotime	Cat# P1045
SDS-PAGE protein loading buffer	Beyotime	Cat# P0015L
Immobilon Western HRP substrate Luminol Reagent	Millipore	Cat# WBKLS0500
Wright-Giemsa Stain Solution	Solarbio	Cat# P1020
**Critical commercial assay**		
TransDirect® Mouse Genotyping Kit	TransGen Biotech Co., Ltd.	Cat# AD501-01

Verheff's Elastic Stain Kit	Abiowell	Cat# AWI0267b
Hifair® AdvanceFast One-step RT-gDNA Digestion SuperMix	Yeasen	Cat# 11151ES60
Veterinary fully automatic hematology analyzer	Mindray	Cat# BC2800vet
Mouse IL1B ELISA Kit	Renjie Biotech	Cat# RJ16944
Mouse TNFA ELISA Kit	Renjie Biotech	Cat# RJ17929
Mouse IL6 ELISA Kit	Abclonal	Cat# RK00008
Mouse CCL2 ELISA Kit	Abclonal	Cat# RK00381
Mouse cAMP ELISA Kit	Jianglai biotech	Cat# JL13362
PKA Kinase Activity Assay Kit	Abcam	Cat# ab139435
2X Universal SYBR Green Fast qPCR Mix	Abclonal	Cat# RK21203
Mouse marrow neutrophil isolation kit	Solarbio	Cat# P8550
**Experimental model**		
C57BL/6J mice	SPF Hunan SJA Laboratory Animal Co., Ltd.	N/A
PI3Kγ-KO-/- mice	GemPharmatech Co. Ltd	Cat# T009406

Table S2: the patient clinical information in the present study. (n=7)

Patient	Gender	Age	Smoking	Diameter(mm)	HL	HTN	CAD
ID1	Male	72	no	43	yes	yes	no
ID2	Female	63	no	42	no	yes	no
ID3	Male	65	yes	89	yes	no	no
ID4	Male	64	yes	43	no	no	no
ID5	Male	77	yes	71	yes	yes	no
ID6	Male	58	yes	40	no	yes	yes
ID7	Male	22	no	68	no	yes	no

HL: Hyperlipidemia; HTN: Hypertension; CAD: Coronary artery disease.

Table S3: The primer sequences used in the present study.

Gene name	Primer Sequence (5'-3')
Pik3cg-WT	Forward: ACGCTGAGCCTTCTCTCCATC
	Reverse: CCATAACCCTGGAAACAATGGC
Pik3cg-KO	Forward: GCTGCGCTGCCTTATTTAACTTT
	Reverse: CTGCGAGAGTTAGGGACTCAGGTGT
Tagln	Forward: CCAACAAGGGTCCATCCTACG
	Reverse: ATCTGGGCGGCCTACATCA
Cnn1	Forward: GACGGGATCATTCTTTGCGAA
	Reverse: CCCCATACTTGGTAATGGCTTTG
Acta2	Forward: GTCCCAGACATCAGGGAGTAA
	Reverse: TCGGATACTTCAGCGTCAGGA
Myh11	Forward: ATGAGGTGGTCGTGGAGTTG
	Reverse: GCCTGAGAAGTATCGCTCCC
Klf4	Forward: GTGCCCCGACTAACCGTTG
	Reverse: GTCGTTGAACTCCTCGGTCT
Spp1	Forward: AGCAAGAAACTCTTCCAAGCAA
	Reverse: GTGAGATTCGTCAGATTCATCCG
IL6	Forward: TACCACTTCACAAGTCGGAGGC
	Reverse: CTGCAAGTGCATCATCGTTGTTC
Ccl2	Forward: GCTACAAGAGGATCACCAGCAG
	Reverse: GTCTGGACCCATTCCTTCTTGG
TNF alpha	Forward: CAGGCGGTGCCTATGTCTC
	Reverse: CGATCACCCCGAAGTTCAGTAG
IL-1β	Forward: GAAATGCCACCTTTTGACAGTG
	Reverse: TGGATGCTCTCATCAGGACAG
Mmp2	Forward: ACCTGAACACTTTCTATGGCTG
	Reverse: CTTCCGCATGGTCTCGATG
Mmp9	Forward: GCAGAGGCATACTTGTACCG
	Reverse: TGATGTTATGATGGTCCCACTTG

Actb	Forward: GTGCTATGTTGCTCTAGACTTCG<br>Reverse: ATGCCACAGGATTCCATACC

Figure S1

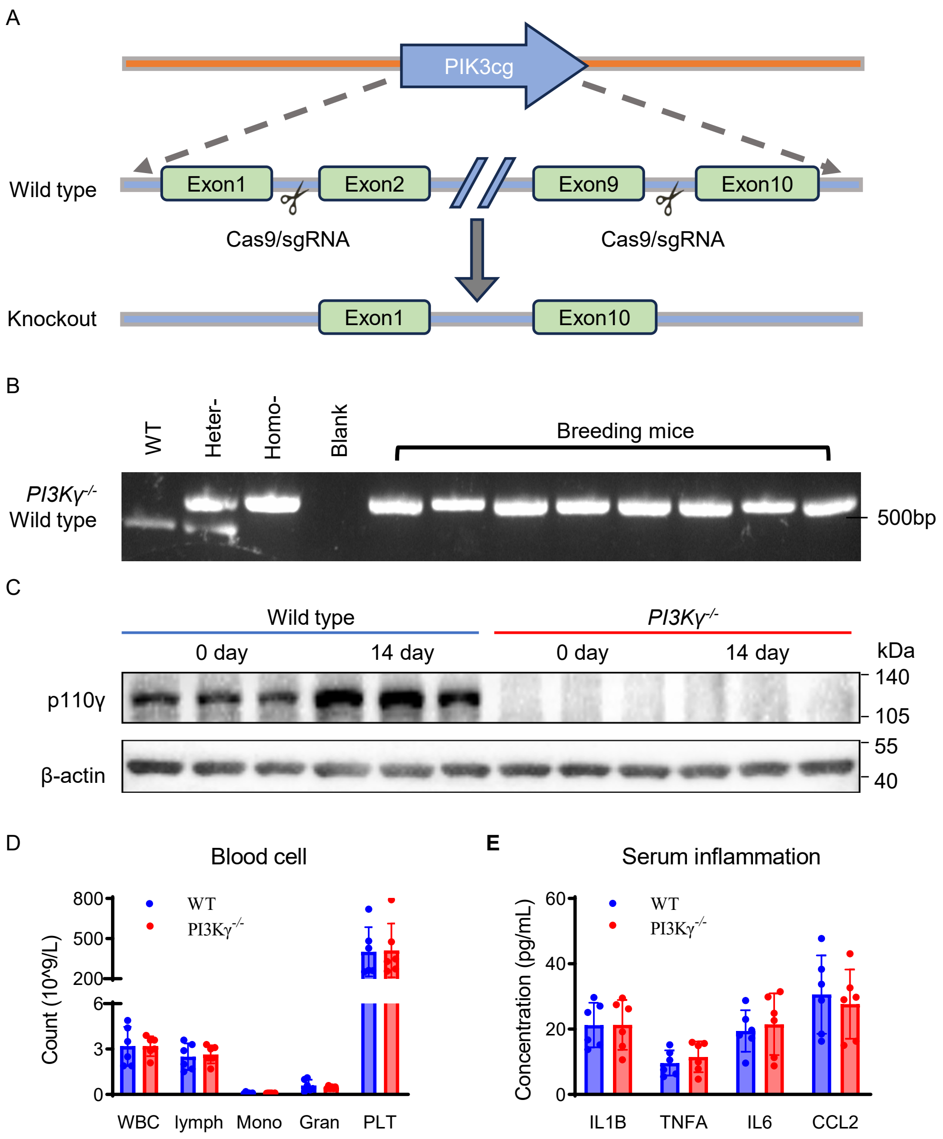

A
PIK3cg
Wild type
Exon1
Exon2
Exon9
Exon10
Cas9/sgRNA
Cas9/sgRNA
Knockout
Exon1
Exon10
B
WT
Heter-
Homo-
Blank
Breeding mice
PI3Kγ-/-
Wild type
500bp
C
Wild type
PI3Kγ-/-
0 day
14 day
0 day
14 day
kDa
p110γ
140
105
β-actin
55
40
D
Blood cell
WT
PI3Kγ-/-
Count (10^9/L)
800
500
200
6
3
0
WBC
lymph
Mono
Gran
PLT
E
Serum inflammation
WT
PI3Kγ-/-
Concentration (pg/mL)
60
40
20
0
IL1B
TNFA
IL6
CCL2

Figure S2

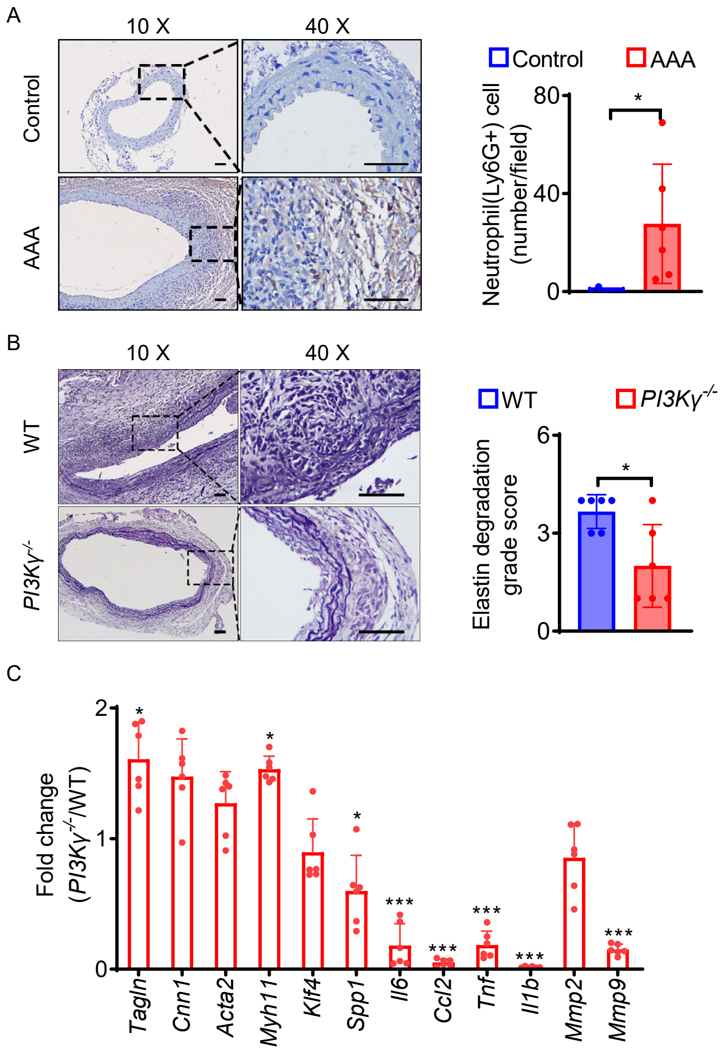

A
10 X
40 X
Control
AAA
Control
AAA
Neutrophil(Ly6G+) cell (number/field)
80
40
0
*
B
10 X
40 X
WT
PI3Kγ-/-
WT
PI3Kγ-/-
Elastin degradation grade score
6
3
0
*
C
Fold change (PI3Kγ-/-/WT)
2
1
0
*
*
*
***
***
***
***
***
Tagln
Cnn1
Acta2
Myh11
Klf4
Spp1
Il6
Ccl2
Tnf
Il1b
Mmp2
Mmp9

Figure S3

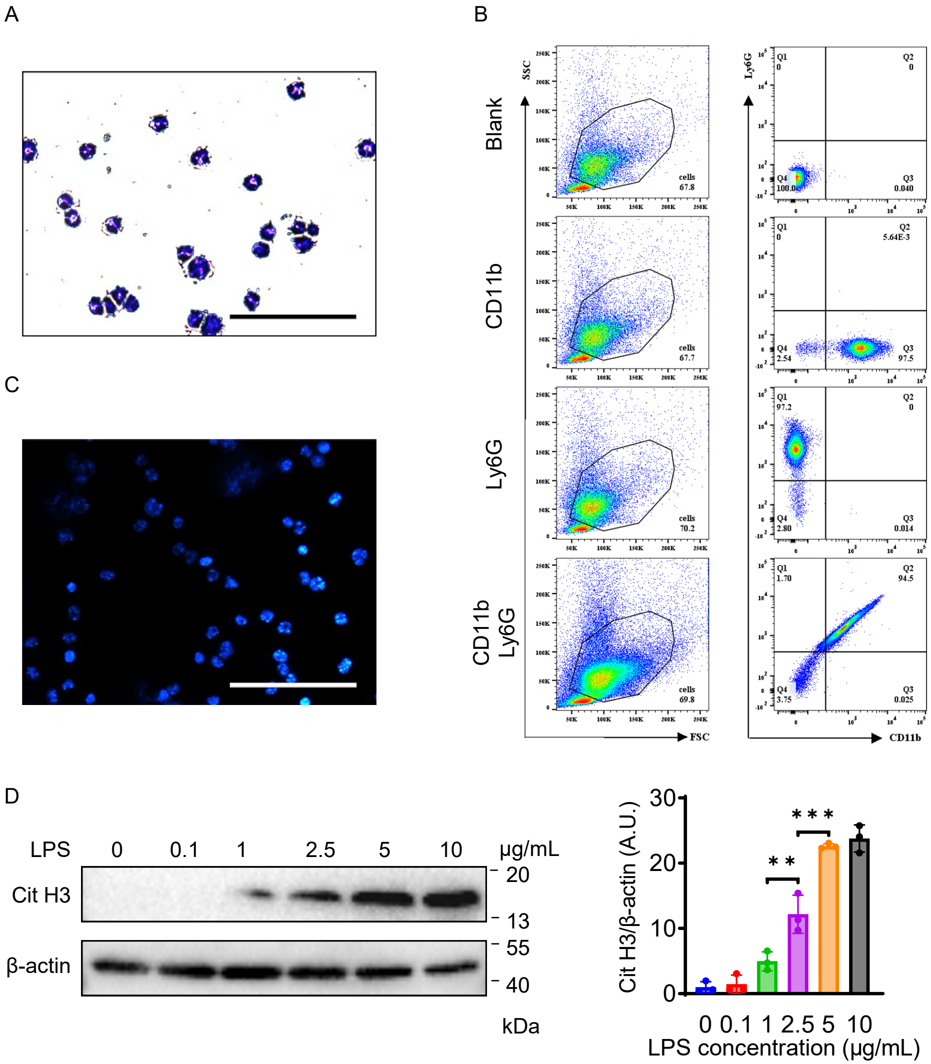
A
B
Blank
CD11b
Ly6G
CD11b Ly6G
SSC
FSC
Ly6G
CD11b
cells 67.8
cells 67.7
cells 70.2
cells 69.8
Q1 0
Q2 0
Q3 0.040
Q4 100.0
Q1 0
Q2 5.64E-3
Q3 97.5
Q4 2.54
Q1 97.2
Q2 0
Q3 0.014
Q4 2.80
Q1 1.70
Q2 94.5
Q3 0.025
Q4 3.75
C
D
LPS 0 0.1 1 2.5 5 10 µg/mL
Cit H3
β-actin
20
13
55
40
kDa
Cit H3/β-actin (A.U.)
0 0.1 1 2.5 5 10
LPS concentration (µg/mL)
**
***

Figure S4

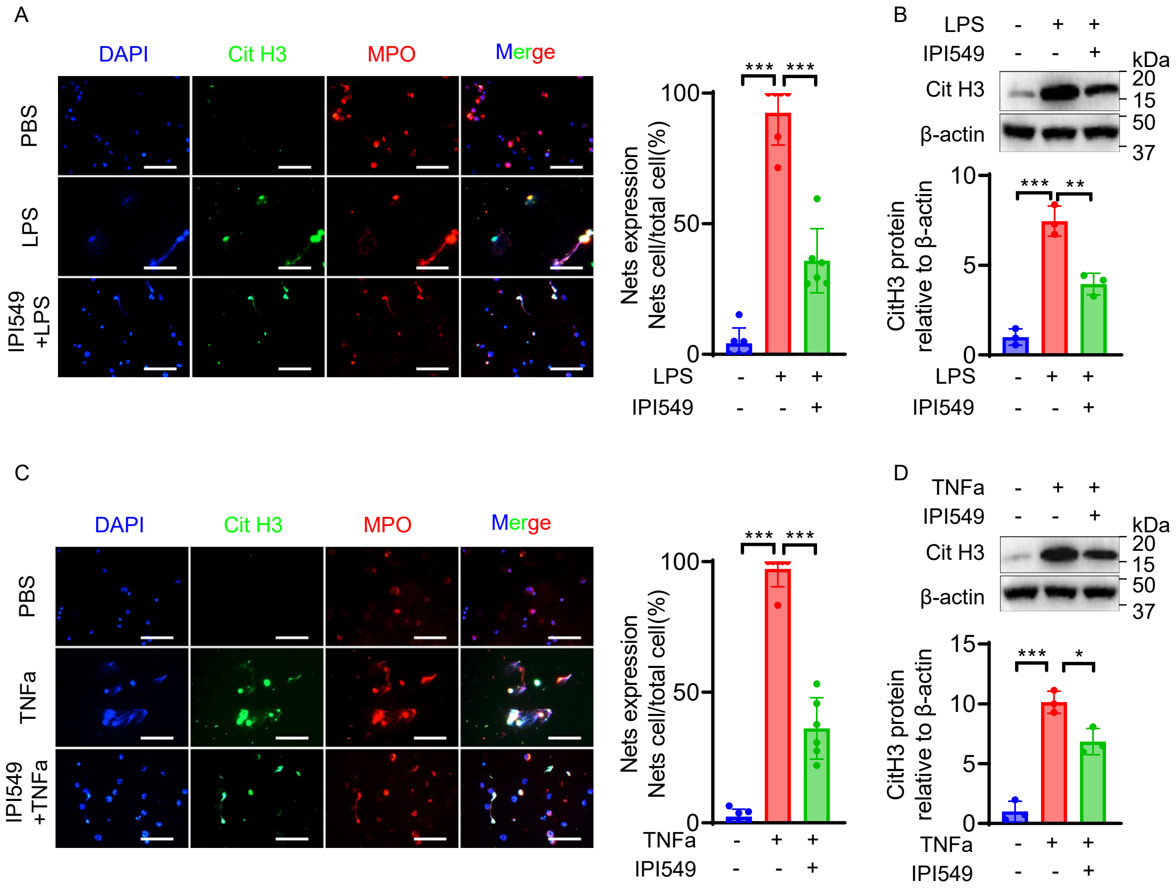
A
DAPI
Cit H3
MPO
Merge
PBS
LPS
IPI549 +LPS
Nets expression
Nets cell/total cell(%)
100
50
0
***
***
LPS - + +
IPI549 - - +
B
LPS - + +
IPI549 - - +
kDa
Cit H3
β-actin
20
15
50
37
CitH3 protein relative to β-actin
10
5
0
***
**
LPS - + +
IPI549 - - +
C
DAPI
Cit H3
MPO
Merge
PBS
TNFa
IPI549 +TNFa
Nets expression
Nets cell/total cell(%)
100
50
0
***
***
TNFa - + +
IPI549 - - +
D
TNFa - + +
IPI549 - - +
kDa
Cit H3
β-actin
20
15
50
37
CitH3 protein relative to β-actin
15
10
5
0
***
*
TNFa - + +
IPI549 - - +

Figure S5

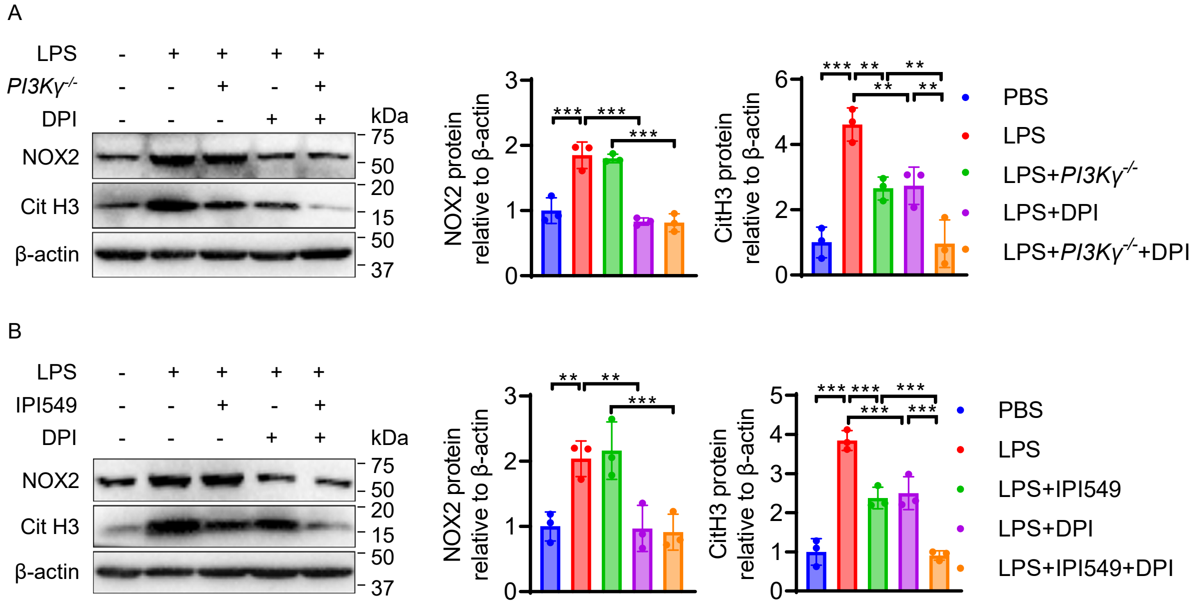

A
LPS
PI3Kγ-/-
DPI
kDa
NOX2
Cit H3
β-actin
75
50
20
15
50
37
NOX2 protein relative to β-actin
CitH3 protein relative to β-actin
PBS
LPS
LPS+PI3Kγ-/-
LPS+DPI
LPS+PI3Kγ-/-+DPI
B
LPS
IPI549
DPI
kDa
NOX2
Cit H3
β-actin
NOX2 protein relative to β-actin
CitH3 protein relative to β-actin
PBS
LPS
LPS+IPI549
LPS+DPI
LPS+IPI549+DPI

Figure S6

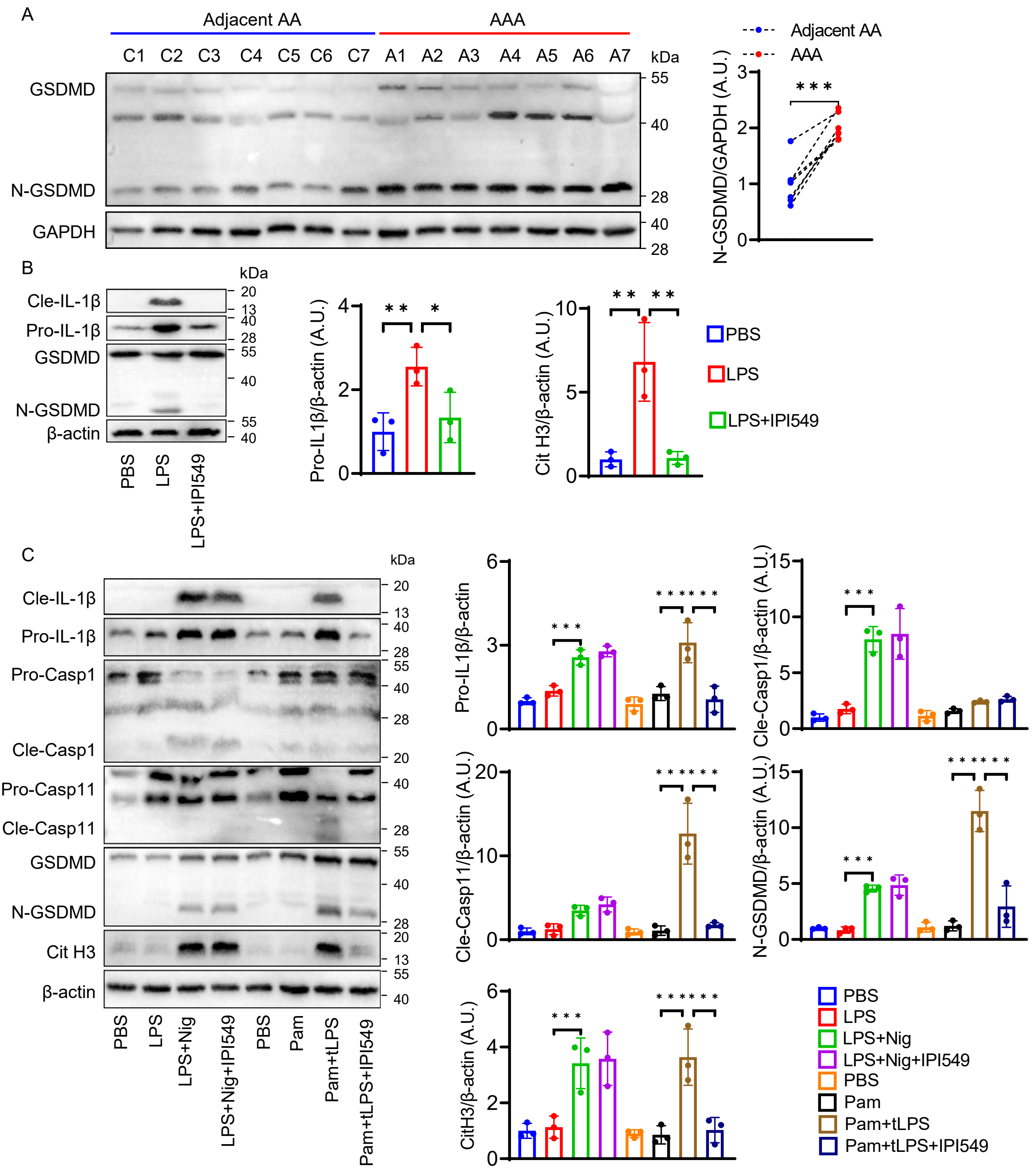
A
Adjacent AA
AAA
C1 C2 C3 C4 C5 C6 C7 A1 A2 A3 A4 A5 A6 A7
kDa
GSDMD
N-GSDMD
GAPDH
55
40
28
40
28
Adjacent AA
AAA
N-GSDMD/GAPDH (A.U.)
***
B
kDa
Cle-IL-1β
Pro-IL-1β
GSDMD
N-GSDMD
β-actin
PBS
LPS
LPS+IPI549
Pro-IL1β/β-actin (A.U.)
Cit H3/β-actin (A.U.)
PBS
LPS
LPS+IPI549
C
Cle-IL-1β
Pro-IL-1β
Pro-Casp1
Cle-Casp1
Pro-Casp11
Cle-Casp11
GSDMD
N-GSDMD
Cit H3
β-actin
PBS
LPS
LPS+Nig
LPS+Nig+IPI549
PBS
Pam
Pam+tLPS
Pam+tLPS+IPI549
Pro-IL1β/β-actin
Cle-Casp1/β-actin (A.U.)
Cle-Casp11/β-actin (A.U.)
N-GSDMD/β-actin (A.U.)
CitH3/β-actin (A.U.)
PBS
LPS
LPS+Nig
LPS+Nig+IPI549
PBS
Pam
Pam+tLPS
Pam+tLPS+IPI549

Figure S7

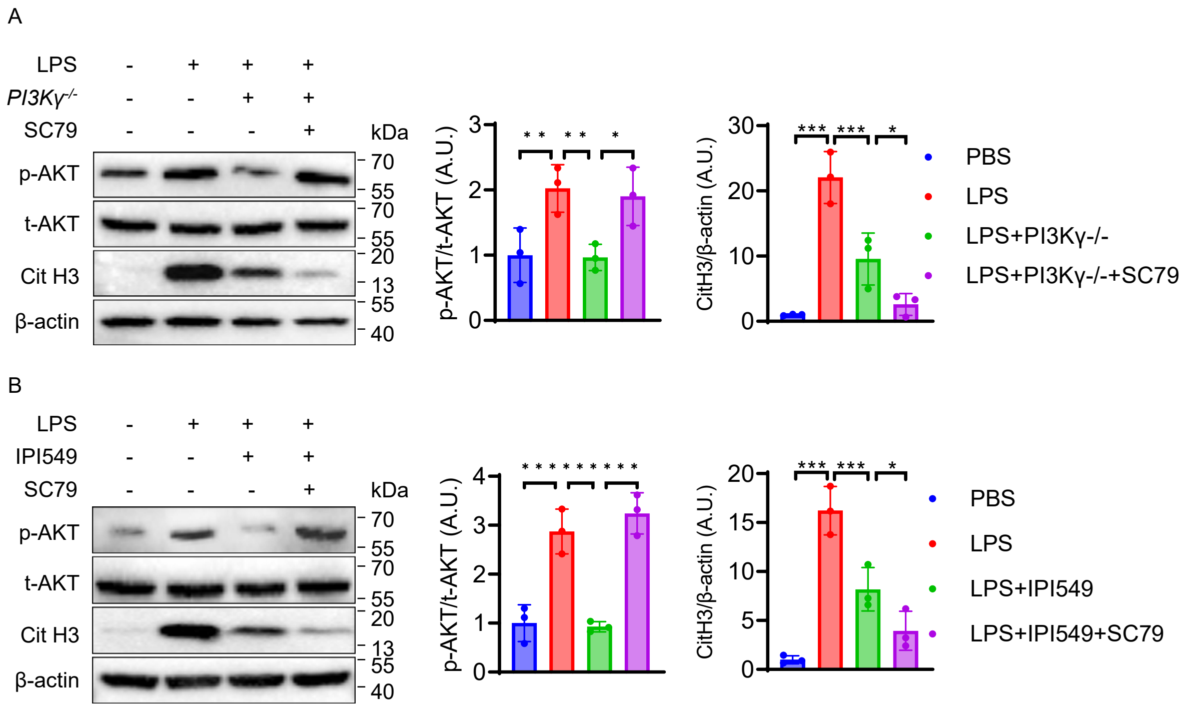
A
LPS
PI3Kγ-/-
SC79
kDa
p-AKT
t-AKT
Cit H3
β-actin
70
55
20
13
40
p-AKT/t-AKT (A.U.)
CitH3/β-actin (A.U.)
PBS
LPS
LPS+PI3Kγ-/-
LPS+PI3Kγ-/-+SC79
B
IPI549
LPS+IPI549
LPS+IPI549+SC79

Figure S8

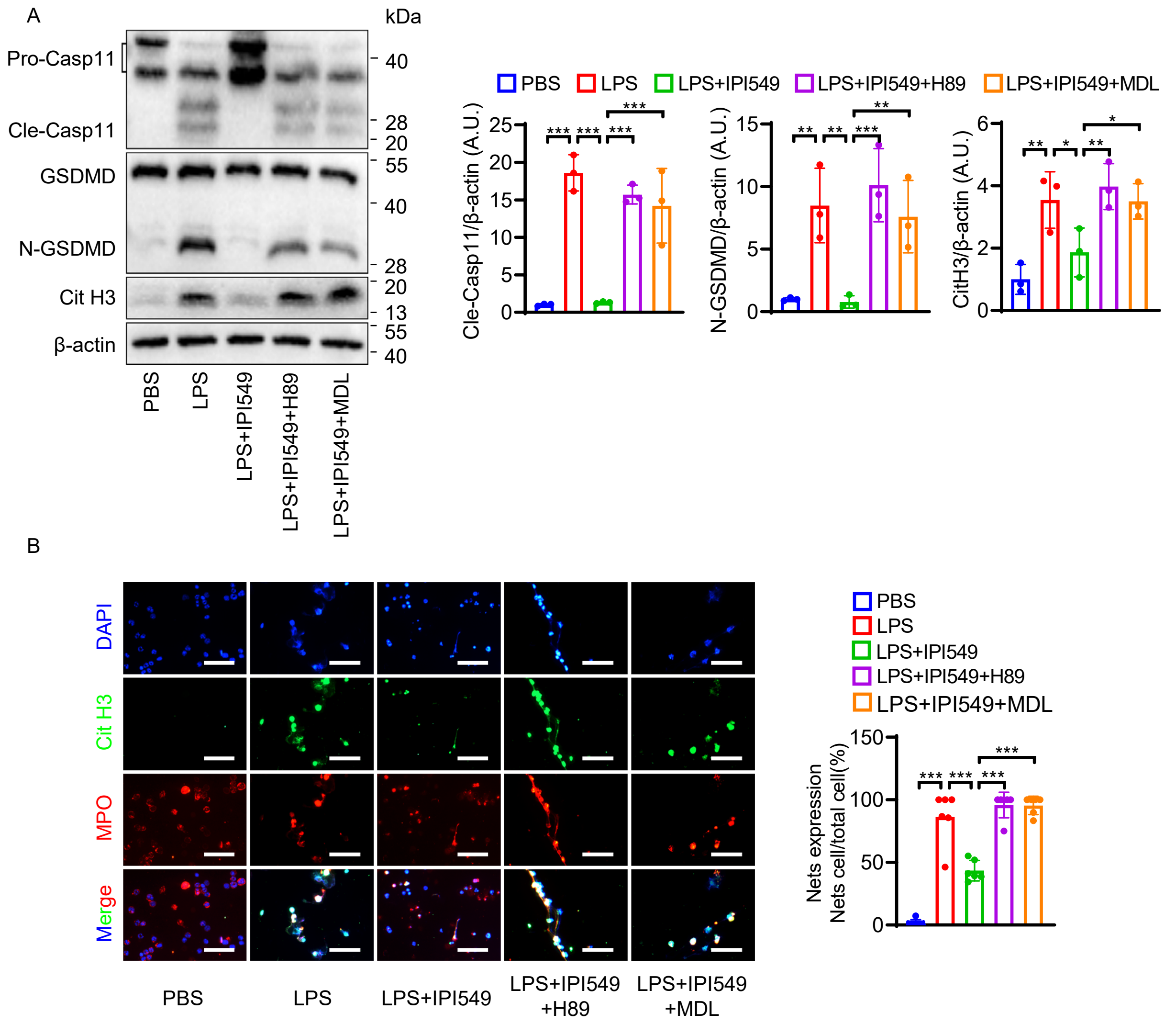

A
kDa
Pro-Casp11
Cle-Casp11
GSDMD
N-GSDMD
Cit H3
β-actin
40
28
20
55
40
28
20
13
55
40
PBS
LPS
LPS+IPI549
LPS+IPI549+H89
LPS+IPI549+MDL
Cle-Casp11/β-actin (A.U.)
N-GSDMD/β-actin (A.U.)
CitH3/β-actin (A.U.)
B
DAPI
Cit H3
MPO
Merge
PBS
LPS
LPS+IPI549
LPS+IPI549 +H89
LPS+IPI549 +MDL
Nets expression
Nets cell/total cell(%)
